# High-resolution quantitative imaging of mammalian and bacterial cells using stable isotope mass spectrometry

**DOI:** 10.1186/jbiol42

**Published:** 2006-10-05

**Authors:** Claude Lechene, Francois Hillion, Greg McMahon, Douglas Benson, Alan M Kleinfeld, J Patrick Kampf, Daniel Distel, Yvette Luyten, Joseph Bonventre, Dirk Hentschel, Kwon Moo Park, Susumu Ito, Martin Schwartz, Gilles Benichou, Georges Slodzian

**Affiliations:** 1 National Resource for Imaging Mass Spectrometry, Harvard Medical School and Department of Medicine, Brigham and Women's Hospital, Cambridge, MA 02139, USA; 2 Cameca, 29 Quai des Gresillons, 92622 Gennevilliers Cedex, France; 3 NSee Inc., 106 Greenhaven Lane, Cary, NC 27511, USA; 4 Torrey Pines Institute for Molecular Studies, San Diego, CA 92121, USA; 5 Ocean Genome Legacy Foundation, Ipswich, MA 01938, USA; 6 Harvard Medical School and Renal Division, Brigham and Women's Hospital, Boston, MA 02115, USA; 7 Harvard Medical School, Boston, MA 02115, USA; 8 Department of Microbiology, University of Virginia, Charlottesville, VA 22908, USA; 9 Harvard Medical School and Department of Surgery, Massachusetts General Hospital, Boston, MA 02114, USA; 10 Universite Paris-Sud, Centre de Spectrométrie Nucléaire et de Spectrométrie de Masse, 91406 Orsay, France

## Abstract

**Background:**

Secondary-ion mass spectrometry (SIMS) is an important tool for investigating isotopic composition in the chemical and materials sciences, but its use in biology has been limited by technical considerations. Multi-isotope imaging mass spectrometry (MIMS), which combines a new generation of SIMS instrument with sophisticated ion optics, labeling with stable isotopes, and quantitative image-analysis software, was developed to study biological materials.

**Results:**

The new instrument allows the production of mass images of high lateral resolution (down to 33 nm), as well as the counting or imaging of several isotopes simultaneously. As MIMS can distinguish between ions of very similar mass, such as ^12^C^15^N^- ^and ^13^C^14^N^-^, it enables the precise and reproducible measurement of isotope ratios, and thus of the levels of enrichment in specific isotopic labels, within volumes of less than a cubic micrometer. The sensitivity of MIMS is at least 1,000 times that of ^14^C autoradiography. The depth resolution can be smaller than 1 nm because only a few atomic layers are needed to create an atomic mass image. We illustrate the use of MIMS to image unlabeled mammalian cultured cells and tissue sections; to analyze fatty-acid transport in adipocyte lipid droplets using ^13^C-oleic acid; to examine nitrogen fixation in bacteria using ^15^N gaseous nitrogen; to measure levels of protein renewal in the cochlea and in post-ischemic kidney cells using ^15^N-leucine; to study DNA and RNA co-distribution and uridine incorporation in the nucleolus using ^15^N-uridine and ^81^Br of bromodeoxyuridine or ^14^C-thymidine; to reveal domains in cultured endothelial cells using the native isotopes ^12^C, ^16^O, ^14^N and ^31^P; and to track a few ^15^N-labeled donor spleen cells in the lymph nodes of the host mouse.

**Conclusion:**

MIMS makes it possible for the first time to both image and quantify molecules labeled with stable or radioactive isotopes within subcellular compartments.

## Background

**Figure 1:**
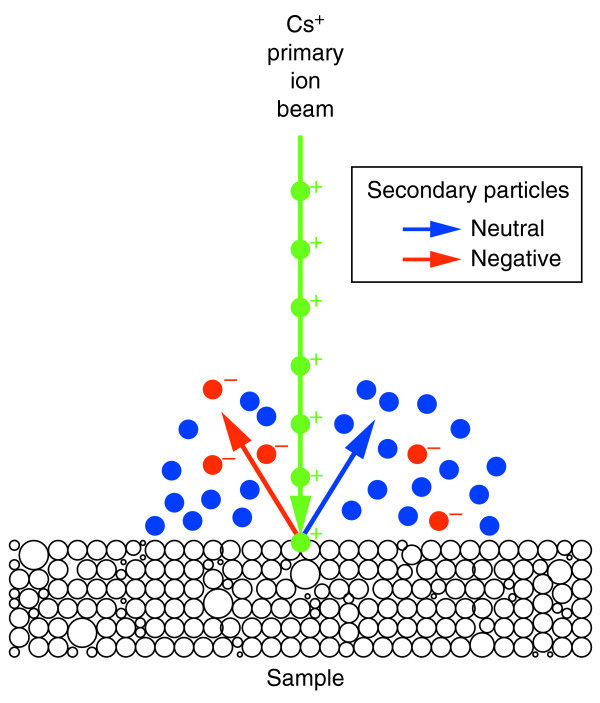
The principle of secondary-ion mass spectrometry. The primary Cs^+ ^beam hits the sample and sputters the surface. Atoms and molecular fragments are ejected from the sample surface; during this process a fraction of the secondary particles are ionized. The identity of the secondary particles, determined by mass spectrometry, indicates the atoms or atomic clusters from the molecules in the sample that have been hit by the primary Cs^+ ^beam. The figure shows only the types of atoms and ions that are relevant to this article; other particles formed by sputtering are not represented. Cs, cesium.

The fundamental discovery that proteins in biological tissues are in a dynamic state was made in the late 1930s using a custom-built mass spectrometer to measure the incorporation into proteins of the stable nitrogen isotope ^15^N [1], which was provided in the mouse diet as ^15^N-leucine and used as a marker of amino acids. These seminal studies could not be pursued at the subcellular level because there was no methodology to simultaneously image and quantitate a stable isotope and because there is no meaningful radioactive isotope of nitrogen. Imaging of stable-isotope distribution has been possible, however, since the development of mass filtered emission ion microscopy using secondary ions by Castaing and Slodzian [2], which is part of the technique later named secondary-ion mass spectrometry (SIMS). With this technique, a beam of ions (the primary-ion beam) is used as a probe to sputter the surface atomic layers of the sample into atoms or atomic clusters, a small fraction of which are ionized (Figure 1) [3]. These secondary ions, which are characteristic of the composition of the region analyzed, can be manipulated with ion optics just as visible light can be with glass lenses and prisms. In a SIMS instrument, the secondary ions are separated according to mass and then used to measure a secondary-ion current or to create a quantitative atomic mass image of the analyzed surface. SIMS has become a major tool in semiconductor and surface-science studies [4], geochemistry [5,6], the characterization of organic material [7], and cosmochemistry [8,9].

Although there has been pioneering work using SIMS in biology [10-14], SIMS technology, until now, has presented irreconcilable tradeoffs [15] that have severely limited its use as a major discovery tool in biomedical research. To make secondary-ion methodology practicable for locating and measuring isotope tags in subcellular volumes, four major issues need to be addressed. First, to produce quantitative ultrastructural images, the technique must have sufficiently high spatial resolution, and quantitation and imaging must be associated. Second, because the quantitation of label involves measuring the excess of an isotope tag above its natural occurrence, and this excess is calculated by the ratio of two isotopes, the data from the two isotopes should be recorded simultaneously, in parallel, and from exactly the same region of the sample (that is, in register). This is to ensure that changes in instrument or sample conditions do not lead to errors in the calculated isotope ratios. Third, because nitrogen has an electron affinity of zero, N^- ^ions do not form; nitrogen must therefore be detected as cyanide ions (CN^-^). Consequently, in order to use the stable isotope ^15^N as a label, the mass resolution of the instrument needs to be high enough to separate the ions ^12^C^15^N^- ^and ^13^C^14^N^-^, which have the same mass number of 27, but do not have exactly the same atomic mass weight: differing by 0.00632 mass unit, less than 1 part in 4,000. Finally, the high mass resolution should not be at the expense of the secondary-ion current; the transmission of secondary ions from sample to detector needs to be high enough to allow data collection from sub-cubic-micrometer volumes and in a reasonable amount of time.

These four requirements necessitate a previously unattainable combination of instrumental capabilities, with the ability to collect large numbers of secondary ions at high mass resolution, parallel detection of several secondary ions, high lateral resolution, and high precision of measurements. A new generation of secondary-ion mass spectrometer has now been developed that can measure several ion masses in parallel, has a high mass resolution (mass/change in mass ratio of approximately 10,000) at high secondary-ion relative transmission (70–80%), and has a high lateral resolution, down to 33 nm [16,17]. In this paper we present some biological applications of this new technology. These extraordinary capabilities allow us, for example, to image and measure in parallel the intracellular distribution of molecules labeled with the stable isotopes ^15^N or ^13^C because we can separate the isobaric species (that is, the species with the same atomic masses): ^12^C^15^N^- ^from ^13^C^14^N^- ^or ^13^C^- ^from ^12^C^1^H^-^. Because the images are produced in parallel from the same sputtered volume they are in exact register with each other and these characteristics are necessary for obtaining quantitative atomic mass images. A quantitative mass image contains at each pixel a number of counts, which are a measure of the selected atomic mass and are directly proportional to the selected atomic mass abundance in the sample, at a location corresponding to the pixel address. Counts from several atomic masses, originating from the same location in the sample, can be recorded in parallel at the same pixel address, which allows us to derive meaningful isotope ratios. Isotope ratios are at the core of the methodology. When the sample has been labeled with a given isotope, a ratio higher than its natural abundance indicates the presence of the marker isotope at a particular location as well as measuring its relative excess. In addition, the high stabilities of the primary beam, the ion optics, the mass spectrometer and the detectors contribute to very precise measurements.

We have developed the use of this new generation of SIMS, together with tracer methods and quantitative image-analysis software, for locating and measuring molecules labeled with stable isotopes in subcellular compartments, a development that we call multi-isotope imaging mass spectrometry (MIMS). In this paper we present a range of examples showing how MIMS can be used to provide atomic mass images of biological specimens, and how in combination with stable isotope labeling it provides qualitative and quantitative information that is not possible to obtain with other methods.

## Results

### Imaging of unlabeled cells and tissue sections

**Figure 2:**
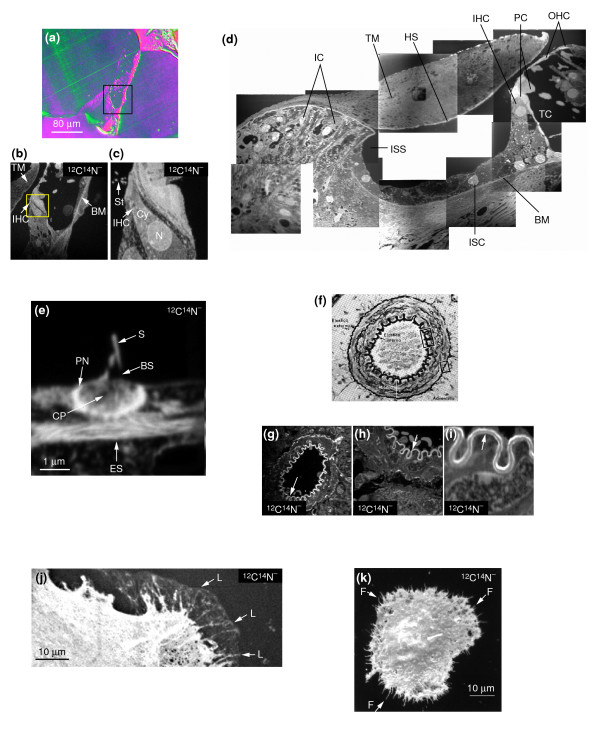
Imaging sections and whole cells with MIMS. **(a-c) **A 0.5-μm epon section of a mouse cochlea mounted on silicon. BM, basilar membrane; Cy, cytoplasm; IHC, inner hair cell; N, nucleus; St, stereocilia; TM, tectorial membrane. (a) Image obtained by reflection differential interference contrast microscopy (RDIC). Scale bar = 80 μm. The boxed area corresponds to the field analyzed with MIMS in (b). (b) MIMS analysis of the same section (80 μm across) at mass ^12^C^14^N; acquisition time 1 min. The boxed area corresponds to the field analyzed at higher resolution in (c). (c) A higher-magnification image of a 20-μm wide part of (b); acquisition time 10 min. **(d) **A mosaic image of a mouse cochlea, compiled from ten individual tiled ^12^C^14^N^- ^mass images. BM, basilar membrane; HS, Hensen's stripe; IC, interdental cells; IHC, inner hair cell; ISC, inner sulcus cell; ISS, inner spiral sulcus; OHC, outer hair cells; PC, pillar cells; TC, tunnel of Corti; TM, tectorial membrane. Acquisition time 2 min per tile. **(e) **High spatial resolution mass image of stereocilia. BS, base of stereocilium; CP, cuticular plate; ES, an elongated structure that is not visible by optical or electron microscopy; PN, pericuticular necklace; S, stereocilium. Scale bar = 1 μm. Conditions of MIMS analysis: beam current 0.4 pA; beam diameter 100 nm; field 6 × 6 μm; 256 × 256 pixels; 18 msec/pixel. For further details see Additional data file 7. **(f) **Reference photomicrograph of a muscular artery from the rat stained with aldehyde-fuchsin. Original magnification 52× [45]. **(g-i) **Contrast formation in an image of a mouse kidney artery. ^12^C^14^N^- ^MIMS images at successively greater magnification, showing a brightly contrasting structure at the location of and with the appearance of the elastica interna. Image sizes: (g) 60 μm; (h) 30 μm; (i) 8 μm. Acquisition times: (g) 1 min; (h) 20 min; (i) 10 min. **(j,k) **Visualizing whole cells. (j) The surface of an untreated endothelial cell (72 μm × 28 μm, 10 min) and (k) endothelial cell after treatment with cytochalasin D (60 μm square, 10 min). L, lamellipodium; F, retraction fibers. Scale bars = (j,k) 10 μm.

One qualitative application of MIMS is high-resolution imaging. Detailed anatomical images can be obtained from unstained, unlabeled samples using the ^12^C^14^N^- ^secondary ion, as shown by the analysis of a section through mouse cochlea (Figure 2a–c). A fixed, unstained section mounted on silicon was first examined using reflection differential interference contrast (RDIC) microscopy (Figure 2a) to select regions of interest that can be retrieved after the sample is hidden inside the SIMS instrument. The mass image of the ^12^C^14^N^- ^ions sputtered from a 80-μm field corresponding to the boxed area in Figure 2a is shown in Figure 2b, and the mass image of the ^12^C^14^N^- ^ions sputtered from a 20-μm subfield of Figure 2b is shown in Figure 2c. The contrast of these mass images provides a very detailed view of the cochlear structures. Only a few atomic layers of the surface of the sample are sputtered using the standard analytical conditions (see Discussion and [Additional data file 1]). Thus, although the method is nominally destructive, we can analyze the same field repetitively, up to a total of tens of hours or hundreds of scans, without observing gross morphological alterations (data not shown). A large area can be imaged relatively quickly in order to select regions of interest for quantitative analysis, as illustrated by the reconstruction of a mouse cochlea shown in Figure 2d. The mass image of ^12^C^14^N^- ^ions is made up of ten tiles, acquired over a total of 20 minutes, with each tile acquired over an area of 100 × 100 μm in 2 minutes. All the cochlear structures one would expect to see [18] are visible, and are easy to identify by comparing them with conventional histological sections.

The high spatial resolution of MIMS is illustrated by its ability to image individual stereocilia, the mechanosensory organelles of the inner hair cells of the cochlea (Figure 2e; the field analyzed was 6 × 6 μm). The various intracellular structures are easy to identify by comparing the image with electron micrographs of the same structure. We estimate that a lateral resolution better than 33 nm can be achieved using a method derived from the 'knife-edge' technique (see [Additional data file 2]). Because the atomic mass image is formed using only a few atomic layers of the sample, the depth resolution (*z *resolution) can be smaller than 1 nm, much better than the resolution that would be provided by an exceptionally thin electron-microscopy section (10 nm). (In this paper, we define depth resolution as the minimum amount of material that needs to be sputtered to obtain an atomic mass image.) Studies of the same sections with both MIMS and electron microscopy – a technique developed for studying cosmic dust [19] – may help provide complementary structural observations.

We do not know from first principles the mechanism of contrast formation in atomic mass images from unstained samples. A striking observation of intense contrast observed with MIMS at mass ^12^C^14^N, shown in Figure 2g–i, may guide inquiries in this field. We were analyzing a mouse kidney when we observed a brightly contrasted structure, a circular 'snake', along the lining of the lumen of an artery. It is likely that this region is the 'elastica interna' as described in histology texts, a tissue layer that is usually visible only after the arterial tissue has been specially stained (Figure 2f). The MIMS images of the artery in Figure 2g–i were obtained without this special staining. The images indicate that this structure produces a high yield of ^12^C^14^N^- ^ions in comparison with the other regions of the artery, and suggests a relationship between the image obtained and molecular composition and density.

MIMS can be used to visualize whole cells as well as sections. These images have a three-dimensional appearance, showing that MIMS can provide scanning atomic or molecular ion mass images of samples with relief, as does scanning electron microscopy. The lamellipodium of a well-spread endothelial cell imaged by MIMS at mass ^12^C^14^N is shown in Figure 2j; it appears as a light, sheet-like structure with darker lines radiating from the cytoplasm to the external border of the lamellipodium. In contrast, if actin polymerization is blocked by cytochalasin D treatment, the ^12^C^14^N mass image of an endothelial cell shows no lamellipodia but only thin, spike-like projections around the cell circumference, which are most probably retraction fibers (Figure 2k).

In conclusion, MIMS atomic mass images of CN^- ^ions in biological samples are highly contrasted, even though they are obtained without any staining. The various structures are easy to identify down to a lateral resolution of approximately 33 nm, and the depth resolution can be as small as a few atomic layers. The largest single field that can be imaged is approximately 140 × 140 μm, but larger fields can be documented quickly by taking a series of images for 1–2 minutes each. There is no machine-specific requirement for the sample except that a vacuum must be sustained. Because MIMS is a surface-analysis method, one can use many kinds of samples: for example, tissue or cell sections embedded in a medium such as epon, or cells cultured directly on a support that can be brought into the analysis chamber and prepared with any usual histological technique. The thickness of the sample is not critical, provided that the electrical charges deposited can be dissipated. The surface analyzed does not have to be flat, and one can obtain SIMS images of three-dimensional samples.

### Quantitative labeling with stable isotopes

Having established that MIMS can be used to obtain atomic mass images of unstained biological objects, this led us to develop the unique feature of MIMS: the quantitative analysis of isotopes within subcellular compartments. We will now discuss how the technique can be used to measure the incorporation of isotopic tracers within compartments of sub-cubic-micrometer volume. To do this, the sample is labeled with stable isotopes such as ^15^N or ^13^C, which are present at much lower levels naturally than their counterpart ^14^N and ^12^C isotopes, and each isotope is then measured to determine whether it is present in an amount exceeding its natural abundance. Stable isotope labeling can be used, for example, to pursue the classic studies of Schoenheimer at the subcellular level [1].

The isotope abundance is measured by recording the secondary-ion currents (counts/time) obtained from a pair of isotopes, for example, ^13^C and ^12^C, calculating the ratio and then comparing it with their natural abundance ratio. In a control sample, which has not received an excess of the tracer isotope, the counts of each isotope are related to each other by their natural abundance. In other words, there will be a count of ^13^C or of ^15^N such that ^13^C/^12^C = 1.12%, or ^15^N/^14^N (measured as ^12^C^15^N/^12^C^14^N) = 0.367%, calculated from the values of their respective natural abundance. This means that when measured in parallel, all the analytical conditions being the same, the ^15^N (^12^C^15^N) count rate will be 272 times lower than the ^14^N (^12^C^14^N) count rate.

#### Quantitative labeling with ^15^N

The goal of our first experiments was to ensure that we could measure ^15^N/^14^N ratios equivalent to their natural abundance from tiny volumes of untreated control sample. Our first analyses were carried out using a stationary cesium (Cs^+^) primary ion beam. We counted in parallel the secondary ions ^12^C^14^N^- ^and ^12^C^15^N^- ^emitted from various areas smaller than 1 square micrometer in control samples of mouse tissues. We measured ^12^C^15^N/^12^C^14^N isotope ratios in control mouse tissue of 0.366% (standard error (SE) = 0.002, *n *= 12) in the cochlea, 0.368% (SE = 0.001, *n *= 14) in the kidney, and 0.368% (SE = 0.001, *n *= 6) in the intestine. These values are not statistically significantly different from the natural terrestrial value of the ^15^N/^14^N isotope ratio, 0.3673% [20]. This proved the feasibility of using this method on biological samples.

We then showed that we could measure the incorporation of a stable isotope label in an ultra-minute volume of biological material, as done for bulk tissue 60 years ago [1]. We fed mice a diet slightly enriched with ^15^N-L-leucine for a sufficient length of time (14 days) to result in total protein renewal in kidney and intestine. The ^15^N/^14^N isotope ratios determined using a stationary primary ion beam at various areas over the samples were equivalent to the ^15^N/^14^N ratio in the diet determined independently by combustion mass spectrometry analysis (intestine 4.45‰, SE = 0.05, *n *= 7; kidney 4.41‰, SE = 0.03, *n *= 12; diet 4.45‰, SE = 0.02, *n *= 7).

Using this method, only one location can be analyzed at a time and its precise position is difficult to ascertain in the absence of an image. With our instrument, we have developed a much more powerful but more complex method of isotope ratio imaging, where the isotope ratios are calculated from quantitative mass images obtained simultaneously from a set of isotopes. A quantitative mass image, as we call it, is the representation of an analyzed field in which each pixel is the address of a register at which the secondary-ion current of an isotope of interest has been recorded during analysis. Up to four secondary-ion currents, representative of four isotopes, can be recorded simultaneously at each pixel address with our instrument, for example ^12^C^-^, ^13^C^-^, ^12^C^14^N^- ^and ^12^C^15^N^-^. A quantitative image of 256 × 256 pixels thus represents a set of (256 × 256 × 4) or 262,144 numbers. We call a group of pixel addresses a 'region of interest', and the first step in quantitation is to extract the values of counts/time/isotope from groups of pixels or from individual pixels. This allows us to measure many more regions from a single analytical field than we could do using a stationary beam, and also to associate quantitation and localization among cells and subcellular domains. All the mass imaging in the rest of this paper will refer to quantitative mass imaging.

**Figure 3:**
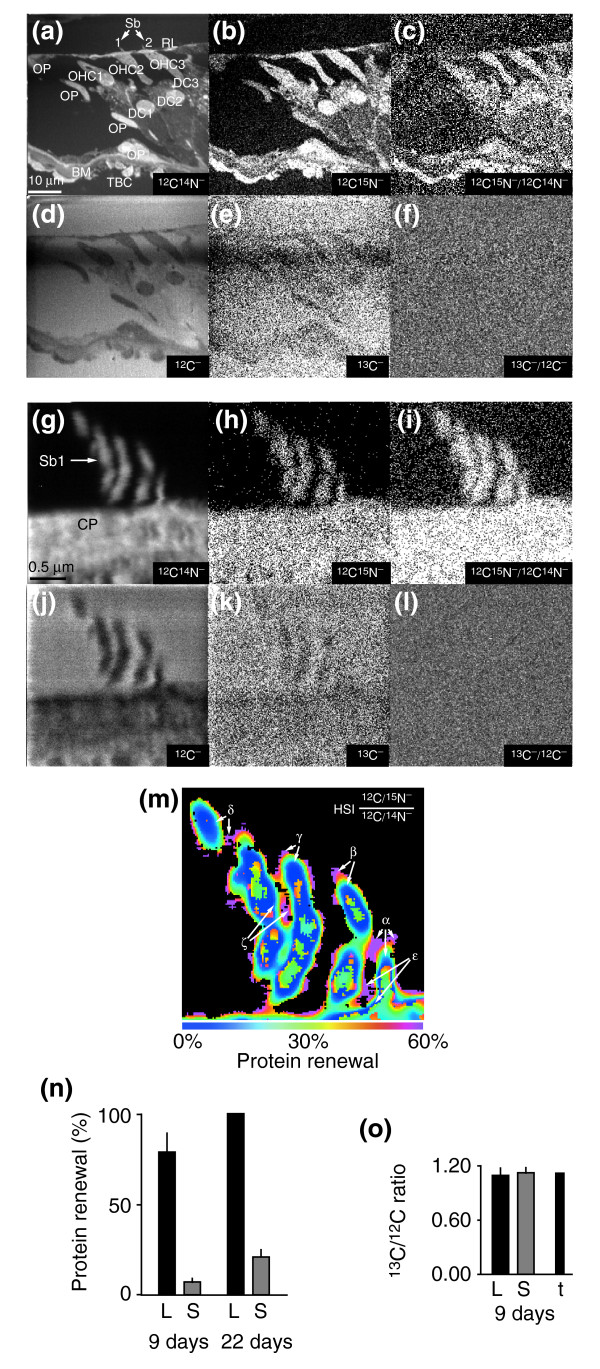
MIMS analysis of stereocilia from mice fed ^15^N-L-leucine. **(a-f) **Quantitative MIMS images of cochlear hair cells from mice after 9 days on the ^15^N-L-leucine diet. DC, Deiter cells; OP, outer pillar cells; RL, reticular lamina; TBC, tympanic border cells (below the basilar membrane); Sb1 and Sb2, stereocilia bundles; other abbreviations are as in Figure 2. All images are 60 × 60 μm (256 × 256 pixels) and have an acquisition time of 10 msec/pixel. (a) ^12^C^14^N^-^, (b) ^12^C^15^N^-^, (c) ^12^C^15^N^-^/^12^C^14^N^- ^ratio image, (d) ^12^C^-^, (e) ^13^C^-^, (f) ^13^C^-^/^12^C^- ^ratio image. The images in (c,f) result from the pixel-by-pixel division of the ^12^C^15^N^- ^image by the ^12^C^14^N^- ^image and of the ^13^C^- ^image by the ^12^C^- ^image, respectively. Scale bar = 10 μm. **(g-l) **High-resolution quantitative MIMS images of the stereocilia labeled Sb1 in (a). The isotopes and ratios shown in each image are indicated and are the same as the equivalent images in (a-f). All images are 3 × 3 μm (256 × 256 pixels) and an acquisition time of 40 msec/pixel. Scale bar = 0.5 μm. **(m) **HSI image of the ^12^C^15^N/^12^C^14^N ratio derived from (h) and (g). The colors correspond to the excess ^15^N derived from the measured ^12^C^15^N^-^/^12^C^14^N^- ^isotope ratios, expressed as a percentage of the ^15^N excess in the feed, which is a measure of protein renewal; values range from 0% (blue) to 60% and higher (magenta). Small magenta areas (α, β, γ, δ, ε, and ζ) indicate excess ^15^N. The image is 3 × 3 μm (256 × 256 pixels) and dwell time was 40 msec/pixel. **(n) **Bar graph of the mean percentage at the stereocilia level of the ^15^N excess in the feed, which is a measure of protein renewal, after 9 days or 22 days of ^15^N-L-leucine diet. L, inter-stereocilia structures; S, core stereocilia at 100–200 nm from L. **(o) **Bar graph of the mean value of the ^13^C/^12^C ratio measured after 9 days at the same locations as in (n). t, value of the natural terrestrial ^13^C/^12^C ratio.

We illustrate quantitative mass imaging of ^15^N with a study of ^15^N-leucine incorporation in the mouse cochlea, a highly organized tissue with several different cell types, and in a subcellular structure of this tissue, the stereocilium, the mechanosensing organelle of hair cells. The secondary-ion mass images of a field of cochlear tissue from a mouse that has been on a ^15^N-L-leucine diet for 9 days are shown in Figure 3a–f. Additional data file 3 describes how the quantitative data are extracted from these images. Mass images of ^12^C^-^, ^13^C^-^, ^12^C^14^N^- ^and ^12^C^15^N^- ^ions were acquired in parallel. The mass image of the ^12^C^14^N^- ^ion (Figure 3a) shows a strikingly detailed histology. ^12^C^14^N^- ^ions arise from nitrogen-containing molecules, the most abundant by far being proteins, which make up 18% of the total weight in most cell types, whereas RNA and DNA make up 1.1% and 0.25%, respectively [21]. The mass image of the ^12^C^15^N^- ^ions (Figure 3b) is similar in form to the ^12^C^14^N^- ^image (Figure 3a) but has much lower counts; the total number of counts of ^12^C^15^N^- ^ions and of ^12^C^14^N^- ^ions are 2.02 × 10^5 ^and 4.52 × 10^7^, respectively (note that the subjective brightness of the images is not directly related to the count rate; see Additional data file 4). The pixel count of the ^12^C^15^N^- ^image is a measure of both natural ^15^N and the supplementary ^15^N arising from the metabolism of ^15^N-L-leucine in the cochlea. This supplementary ^15^N may vary from a minimum of zero to a maximum value equivalent to the ^15^N added to the diet. The image of the internal control ^12^C^- ^(Figure 3d) has a relatively poor contrast compared with the ^12^C^14^N^- ^image (Figure 3a) because a larger fraction of the ^12^C^- ^ions arise from the embedding medium, which has a high and uniform carbon content. The image of the ^13^C^- ^ions (Figure 3e) is similar to the ^12^C^- ^image, but with a much lower count rate; the total number of counts of ^13^C^- ^and of ^12^C^- ^are 2.56 × 10^5 ^and 2.33 × 10^7^, respectively. The pixel counts of the sample resulting in the ^13^C^- ^ion image contain a mean of 1.10% of the ^12^C counts, corresponding to the natural ratio of ^13^C/^12^C.

The ratio images ^12^C^15^N^-^/^12^C^14^N^- ^(Figure 3c) and ^13^C^-^/^12^C^- ^(Figure 3f) result from the pixel-by-pixel division of the ^12^C^15^N^- ^image by the ^12^C^14^N^- ^image and of the ^13^C^- ^image by the ^12^C^- ^image, respectively. The contrast observed in the ^12^C^15^N^-^/^12^C^14^N^- ^image is due to the excess of ^15^N in the area of the cochlea that has incorporated ^15^N derived from the ^15 ^N-L-leucine. The internal control ^13^C^-^/^12^C^- ^ratio image has no contrast because, in the absence of added ^13^C, the value of the ratio is equivalent to the natural ratio in any part of the analyzed field.

Using the quantitative images and the derived ratio images, and guided by the hue saturation intensity (HSI) transformation (see Materials and methods), we can now calculate a value for ^15^N incorporation into the main structures shown in Figure 3a–f. This can be expressed as percentage renewal by comparing the excess ^15^N in the tissue with the excess ^15^N in the diet (see Materials and methods). These values represent overall protein renewal among the different cochlear structures, as demonstrated for whole tissue in the classic work of Schoenheimer [1]. After 9 days of a ^15^N-L-leucine diet, the incorporation of ^15^N is markedly different among specific cell types. The outer hair cells have a ^15^N renewal of 52.5% ± 1.8 SD, not significantly different from that of the Deiter cells (47.2% ± 4.8 SD), or of the proximal part of the outer pillar cell above the basilar membrane (46.0% ± 5.7 SD), and of one population of tympanic border cells (48.3% ± 1.5 SD). The basilar membrane has a small ^15^N renewal (overall 21.1% ± 6.0 SD), not statistically different from part of the outer pillar cells at the level of the Deiter cells (18.4% ± 3.7 SD). Overall, the reticular lamina has a ^15^N renewal of 30.8% ± 8.9 SD, significantly higher than the basilar membrane and the distal outer pillar cells, and significantly lower than that of the outer hair cells. The lone outer hair-cell nucleus observed has a ^15^N renewal of 35.5%. Finally, we measured a second population of tympanic border cells with a ^15^N renewal significantly greater than in any other area (72.8% ± 2.5 SD). The internal control provided by the epon embedding medium had a ^12^C^15^N/^12^C^14^N isotope ratio of 0.365% ± 0.089 SD, equivalent to the natural abundance ratio and corresponding to a ^15^N renewal of 0%.

The unique power of MIMS is demonstrated by the quantitative imaging of subcellular structures at high resolution, revealing sub-cubic-micrometer-sized zones with high ^15^N renewal, and thus probably high protein renewal. In addition, the experiment showed that the same sample can be analyzed repetitively at a variety of spatial resolutions. We analyzed one of the bundles of stereocilia (Sb1, indicated by a white arrow in Figure 3a) at high resolution; we used a field of 3 × 3 μm, a beam size of about 35 nm, and 256 × 256 pixels (Figure 3g–l). Mass images of the ^12^C^-^, ^13^C^-^, ^12 ^C^14^N^- ^and ^12^C^15^N^- ^ions were acquired in parallel. The ^12^C^14^N^- ^image (Figure 3g) shows one bundle of stereocilia and a fraction of the cuticular plate of one hair cell, barely visible in the lower-resolution image in Figure 3a.

As in the cochlear analysis, but at a subcellular level, the ^12^C^15^N^- ^image (Figure 3h) is similar in form to the ^12^C^14^N^- ^image (Figure 3g) but has much lower counts; the total number of counts of ^12^C^15^N^- ^and of ^12^C^14^N^- ^are 8.36 × 10^4 ^and 1.61 × 10^7^, respectively. The ^12^C^- ^image (Figure 3j) has relatively poor contrast compared with the ^12^C^14^N^- ^image (Figure 3g), as most of the ^12^C^- ^ions arise from the embedding medium. The ^13^C^- ^image (Figure 3k) is similar to the ^12^C^- ^image, but with a much lower count; the total number of counts for ^13^C^- ^and ^12^C^- ^are 4.80 × 10^5 ^and 4.36 × 10^7^, respectively. The pixel counts from the ^13^C^- ^image include the fraction of ^13^C related to the ^12^C content by the natural ratio of ^13^C/^12^C. The ratio images ^12^C^15^N^-^/^12^C^14^N^- ^(Figure 3i) and ^13^C^-^/^12^C^- ^(Figure 3l) result from the pixel-by-pixel division of the ^12^C^15^N^- ^image by the ^12^C^14^N^- ^image and of the ^13^C^- ^image by the ^12^C^- ^image, respectively. The contrast observed in the ^12^C^15^N^-^/^12^C^14^N^- ^image is due to the excess of ^15^N in the stereocilia, cuticular plate, and hair-cell areas that have incorporated ^15^N derived from the ^15^N-L-leucine. The internal control ^13^C^-^/^12^C^- ^ratio image (Figure 3l) has no contrast, as in Figure 3f.

An HSI transformation of the ^12^C^15^N^-^/^12^C^14^N^- ^ratio image of the stereocilia bundle in Figure 3i is shown in Figure 3m. The colors indicate the fractional excess ^15^N derived from the measured ^12^C^15^N^-^/^12^C^14^N^- ^isotope ratios. The HSI image reveals small areas of high excess ^15^N located towards the tips of stereocilia or between stereocilia (magenta); within the stereocilia, close to these areas, there is minimal or no excess ^15^N, as indicated by the predominantly blue-green to blue color.

**Figure 5:**
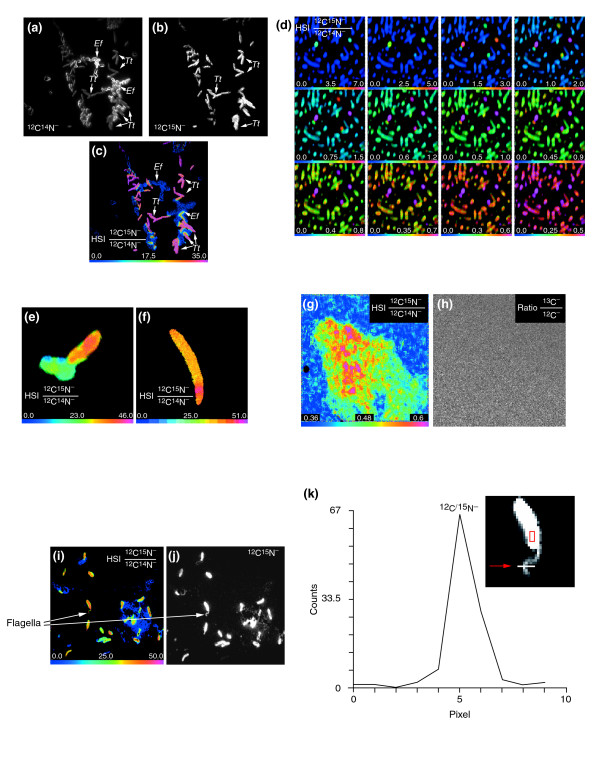
Use of MIMS to study nitrogen-fixing bacteria. **(a-c) **Secondary ion images from the molecular ions (a) ^12^C^14^N^-^, (b) ^12^C^15^N^-^, and (c) the HSI ^12^C^15^N^-^/^12^C^14^N^- ^ratio of a sample containing both *Teredinibacter turnerae *(*Tt*; rod-like cells) and *Enterococcus faecalis *(*Ef*; bunches of rounded cells) cultured in a ^15^N atmosphere for 120 h. Field: 46 × 46 μm (512 × 512 pixels); acquisition time 3 min. The magenta color of the *T. turnerae *cells is an indication of their incorporation and fixation of ^15^N (see Figure 3 for explanation). **(d) **The effect of scaling of the HSI ^12^C^15^N^-^/^12^C^14^N^- ^ratio image (the numerator has been multiplied by 100) from *T. turnerae *cells exposed to a ^15^N atmosphere for 32 h. Assigning the hue spectrum to the whole range of ratio values allows easy identification of bacteria most highly enriched in ^15^N (the turquoise cells in the top left panel). Compressing the hue scale (shown gradually from top left to lower right) causes images of some of the cells to saturate at the magenta level and allows us to easily recognize a succession of cells also enriched in ^15^N, although at a lower level. The isotope values start with 0–7 (top left; a value of 7 is 19-fold higher than the natural ratio) and go to 0–0.5 (bottom right; a value of 0.5 is 1.43 times the natural ratio). The field of view is 13 × 13 μm (256 × 256 pixels); acquisition time 20 min. **(e,f) **HSI image of the ^12^C^15^N^-^/^12^C^14^N^- ^ratio (the numerator has been multiplied by 100) of a *T. turnerae *cell exposed to a ^15^N atmosphere for 96 h. Field: (e) 8 × 8 μm; (f) 6 × 6 μm. Acquisition time: (e) 10 min; (f) 40 min. **(g,h) **HSI image of (g) the ^12^C^15^N^-^/^12^C^14^N^- ^ratio (the numerator has been multiplied by 100) and (h) the ^13^C^-^/^12^C^- ^ratio of *T. turnerae *in shipworm gill bacteriocytes incubated in the presence of a ^15^N atmosphere for 4 h. Field: 10 μm × 10 μm (256 × 256 pixels); acquisition time 60 min. **(i,j) **HSI image (i) of the ^12^C^15^N^-^/^12^C^14^N^- ^ratio (the numerator has been multiplied by 100) and (j) at ^12^C^15^N^- ^of *T. turnerae *exposed for 96 h in a ^15^N atmosphere. Arrows indicate the flagella of the bacteria. Field: 60 × 60 μm (256 × 256 pixels); acquisition time 20 min. **(k) **Line scan across the flagellum observed in (i,j) showing ^12^C^15^N^- ^secondary-ion counts as a function of pixel address across the flagellum. One pixel is equivalent to 234 nm. Inset: arrow points to the flagellum; the red box indicates the area of the bacterium that was used to evaluate the mean ^12^C^15^N^- ^counts.

Guided by the HSI image, we have calculated the values of the ^12^C^15^N^-^/^12^C^14^N^- ^ratios and of the percentage ^15^N renewal for the areas indicated α to ζ and at 100 to 200 nm away from them within the stereocilia core over an approximately equivalent area (Figure 3m and Table 1). We measured high ^15^N renewal in areas α to ζ (79.4% ± 12.7 SE, *n *= 5), whereas at 200 nm away the ^15^N renewal in stereocilia was very low (4.6% ± 1.27 SE, *n *= 5). Finally, MIMS allowed us to estimate from the relative counting of mass ^12^C^14^N in areas α to ζ and in stereocilia that the above values may have been produced by objects about 5 nm wide (see also Figure 5k below in the section entitled 'Quantitative labeling of prokaryotic with gaseous ^15^N). The overall mean values of ^15^N renewal in structures between stereocilia, found with HSI, and in adjacent stereocilium cores are shown in Figure 3n. After 9 days on the ^15^N-L-leucine diet, the mean ^15^N incorporation into the inter-stereocilia structures was 78.6% ± 10.1 SE (*n *= 7). In the adjacent stereocilium core (200 nm away), the ^15^N incorporation was 7.1% ± 2.1 SE (*n *= 7). After 22 days on the ^15^N-L-leucine diet, the incorporation of ^15^N into the inter-stereocilia structures was 100% of its content in the diet, and in the adjacent stereocilium cores, ^15^N incorporation was 20.9% ± 3.8 SE (*n *= 4). In the areas in which ^15^N values were very different, the internal control ^13^C/^12^C ratios (Figure 3o) were very similar between inter-stereocilia structures (1.09% ± 0.04 SE, *n *= 7) and adjacent stereocilium cores (1.12% ± 0.03 SE, *n *= 7), and are statistically equivalent to the natural terrestrial ratio of 1.12% [20].

**Table 1:** Calculated percent nitrogen renewal from stereocilia regions analyzed in Figure 3m

	Tip or lateral links			Stereocilia		
		
Location	Pixels (*n*)	% Renewal	√ Area (Å)	Pixels (*n*)	% Renewal	√ Area (Å)
α_1_	15	100	453	14	4.9	438
α_2_	13	100	423	12	8.0	406
β	33	45.0	672	24	6.0	573
γ	10	52.0	370	23	0.37	562
δ	13	100	422	15	3.6	453
ε	36	54.9	703	41	12.2	750
ζ	17	40.0	483	24	5.0	574

We can thus measure with high precision in a single analyzed field a variety of values of ^15^N incorporation among different cell types, as calculated from the quantitative mass images in Figure 3a–c, or among subcellular structure over an area of 9 μm^2 ^square, as calculated from the quantitative mass images in Figure 3g–i.

#### Quantitative labeling with ^13^C

Despite the importance of free fatty acids (FFAs) for life, studies of their transport are difficult to extend to the cellular scale because no suitable methodology is available. Autoradiography cannot provide quantitative information on accumulation of FFAs in intracellular fat droplets, and fluorescently labeled FFAs may not accurately reflect the transport and metabolism of native FFAs [22,23]. As a result, the mechanism that transports FFAs across a cell membrane remains uncertain (for recent reviews see [24-27]). Using quantitative mass imaging with MIMS we have directly studied the accumulation of ^13^C in cultured adipocytes incubated with ^13^C-labeled oleic acid (^13^C-OA; see [28] for further details). We measured a high level of ^13^C accumulation in intracellular lipid droplets. Quantitative MIMS images were obtained in parallel for ^12^C^-^, ^13^C^-^, ^12^C^14^N^-^, and the isobars ^13^C^14^N^- ^and ^12^C^15^N^-^. The relative excess of ^13^C was measured at three different locations: outside the cell, inside the cell but outside the lipid droplets, and inside the lipid droplets.

**Figure 4:**
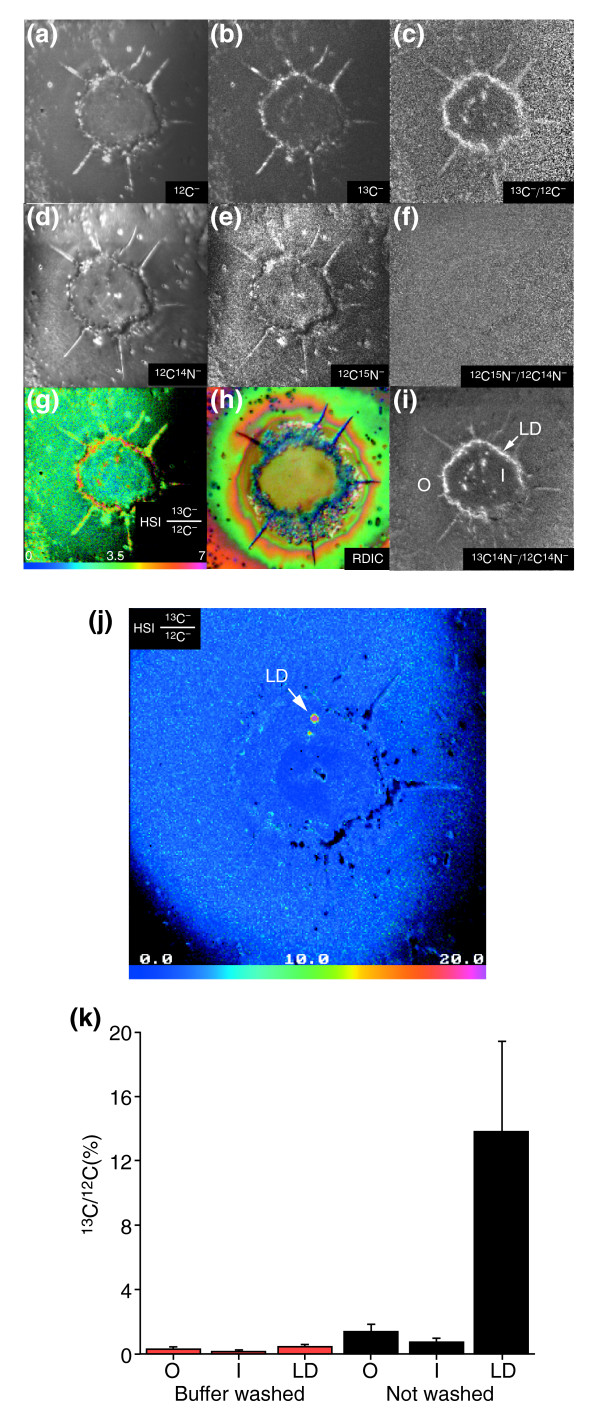
Fatty-acid transport in cultured adipocytes. **(a-i) **MIMS mass images of cells dried with argon after unwashed 3T3F442A adipocytes were incubated with ^13^C^- ^oleate. Images show (a) ^12^C^-^, (b) ^13^C^-^, (d) ^12^C^14^N^-^, and (e) ^12^C^15^N^-^, and their respective ratio images of (c) ^13^C^-^/^12^C^- ^and (f) ^12^C^15^N^-^/^12^C^14^N^-^. (g) HSI image of the ^13^C^-^/^12^C^- ^ratio (the numerator has been multiplied by 100); (h) an RDIC image of the same cells before analysis with MIMS. RDIC images (500×) were obtained using a Nikon Eclipse E800 upright microscope. (i) The ^13^C^14^N^-^/^12^C^14^N^- ^distribution also reveals the excess ^13^C in the lipid droplets. O, outside the cells; I, inside but not in visible lipid droplets; LD, inside the lipid droplets. The MIMS images are 60 × 60 μm (256 × 256 pixels) and were acquired in 40 min. **(j) **HSI of the ^13^C/^12^C ratio after 'shaving' (see text) the adipocyte shown in (a-i); the adipocyte had been exposed to a high primary-ion beam current approximately 1,000-fold more intense than for the previous analysis to quickly remove material from the sample surface in order to analyze deeper within the cell. Field: 60 × 60 μm (256 × 256 pixels); acquisition time 10 msec/pixel. **(k) **Bar graph of the mean and standard deviation values of the ^13^C^-^/^12^C^- ^ratio in 3T3F442A adipocytes. O, outside the cells; I, inside but not in visible lipid droplets; LD, inside the lipid droplets. ^13^C^-^/^12^C^- ^ratio values are shown after subtraction of the natural abundance ratio (1.2%). Adapted with permission from [28].

The quantitative mass images of an adipocyte exposed to ^13^C-OA for 20 minutes are shown in Figure 4a–i. Images of the ^12^C^-^, ^13^C^-^, ^12^C^14^N^- ^and ^12^C^15^N^- ^ions or of the ^12^C^-^, ^13^C^-^, ^12^C^14^N^- ^and ^13^C^14^N^- ^ions were acquired in parallel. Images of the ^12^C^- ^and ^12^C^14^N^- ^ions (Figure 4a,d) show the cell histology. The mass image of the ^13^C^- ^ion (Figure 4b) is similar to the ^12^C^- ^ion image (Figure 4a) in form, but has a lower count rate. The pixel counts of the ^13^C^- ^image include both the natural ^13^C and the supplementary ^13^C from the ^13^C-OA transported into the cell. This supplementary ^13^C is at a maximum at the intracellular lipid droplets, where FFAs accumulate. The mass image of the internal control ^12^C^15^N^- ^ions (Figure 4e) is similar to the ^12^C^14^N^- ^mass image, yet with a much lower count rate. Each pixel of the sample resulting in the ^12^C^15^N^- ^image contains the fraction of ^15^N related to the ^14^N content by the natural ratio of ^15^N/^14^N. The enhanced contrast observed in the ^13^C^-^/^12^C^- ^image (Figure 4c) is due to the excess ^13^C incorporated into the lipid droplets from the transported ^13^C-OA. The internal control ^12^C^15^N^-^/^12^C^14^N^- ^ratio image (Figure 4f) has no contrast because in the absence of added ^15^N, the value of the ratio of ^15^N/^14^N is equivalent to the natural ratio across the analyzed field.

The HSI image of the ^13^C^-^/^12^C^- ^ratio is shown in Figure 4g, and the same cell photographed by RDIC microscopy on the silicon chip before analysis with MIMS is shown in Figure 4h. The ^13^C^-^/^12^C^- ^ratio, indirectly measured as the cyanide ion, ^13^C^14^N^-^/^12^C^14^N^-^, is shown in Figure 4i; this also shows accumulation of ^13^C^- ^in the droplets. In contrast to the high ^13^C^-^/^12^C^- ^ratios found in cells that were incubated with ^13^C-OA, cells washed with buffer solution after ^13^C-OA incubation had low ^13^C^-^/^12^C^- ^ratios (images not shown). In cells not treated with ^13^C-OA, the value of the ^13^C^-^/^12^C^- ^ratio measured under the same conditions was 1.15 ± 0.10%, not significantly different from the terrestrial ^13^C/^12^C value of 1.12%. An indication of the accuracy of these values was obtained from measurements of the ^12^C^15^N^-^/^12^C^14^N^- ^ratio, whose value was 0.36 ± 0.01% in both washed and unwashed cells, in excellent agreement with the natural abundance of 0.37%. The cumulative values obtained from quantitative MIMS atomic mass images and extracted from the isotope ratio images are shown in Figure 4k.

We can remove material quickly from the sample surface in order to study a variety of depths within the cell. We refer to this as 'shaving' the sample. It is accomplished in conditions that give a high primary-ion beam current (such as by removing the objective diaphragm; see Figure 13 in the Discussion section). The results of such shaving are shown in Figure 4j. The adipocyte analyzed in Figure 4a–i was shaved using, for a few minutes, a primary beam current approximately 1,000-fold more intense than for the previous analysis. This uncovered a lipid droplet deeper in the cell with a very high ^13^C^-^/^12^C^- ^ratio, as shown in the HSI image (Figure 4j). Finally, MIMS allows us to acquire hundreds of atomic mass image planes successively from the same cell, opening the door to full three-dimensional volume rendering. We have begun using this capability to study the distribution of ^13^C among the lipid droplets located within a single adipocyte after incubation with ^13^C-OA [29]. In conclusion, MIMS can be used to investigate lipid metabolism with high spatial and quantitative resolution [28]. Unlike other techniques, MIMS allows us to trace and to measure the movement of native FFAs at specific subcellular locations.

#### Quantitative labeling of prokaryotic cells with gaseous ^15^N

The ability to image and measure stable isotopes makes it easy and safe to apply MIMS to samples labeled with a gaseous precursor. Here we describe the application of MIMS to the study of nitrogen fixation in bacteria (Figure 5a–k). *Teredinibacter turnerae *is a diazotrophic (nitrogen-fixing) marine bacterium that can be isolated from the tissues of wood-boring marine bivalves (family Teredinidae) and grown in pure culture [30,31]. *Enterococcus faecalis *is a bacterium that does not fix nitrogen. Both were cultured for 120 hours in a ^15^N atmosphere. Mass images of the ^12^C^-^, ^13^C^-^, ^12^C^14^N^- ^and ^12^C^15^N^- ^ions were acquired in parallel. *T. turnerae *is barely visible at mass ^12^C^14^N^- ^(Figure 5a) but is seen as intensely labeled at mass ^12^C^15^N^- ^(Figure 5b) because it has used gaseous ^15^N to build its molecular constituents. By contrast, *E. faecalis *is visible at mass ^12^C^14^N^- ^(Figure 5a) but not at mass ^12^C^15^N^- ^(Figure 5b) because it does not use gaseous nitrogen and therefore has not incorporated ^15^N above the natural ratio. The HSI image of the ^12^C^15^N^-^/^12^C^14^N^- ^ratio (Figure 5c) shows *E. faecalis *with an isotope ratio equivalent to the natural isotope ratio (blue) and *T. turnerae*, which has incorporated an enormous quantity of ^15^N, with an isotope ratio at least 100 times higher (magenta).

MIMS can also be used to study the distribution of isotope tag incorporation within a bacterial population. The heterogeneity of nitrogen fixation among a population of *T. turnerae *is shown in Figure 5d, where the same field cultured for 32 hours in a ^15^N atmosphere is shown as a series of HSI ^12^C^15^N^-^/^12^C^14^N^- ^ratio images. The different HSI panels reveal the level of ^15^N incorporation in bacteria using a compressed color scale as described in the legend to Figure 5. This analysis reveals the location and the distribution of ratio values, in other words of nitrogen fixation, among the bacteria within the analyzed field. Large differences in the amounts of ^15^N incorporation by *T. turnerae *cultured for 96 hours in a ^15^N atmosphere are demonstrated by the HSI images of the ^12^C^15^N^-^/^12^C^14^N^- ^ratio (Figure 5e,f). Differences are visible among a few bacteria in contact with each other (Figure 5e) and even within a single bacterium (Figure 5f).

MIMS can detect and measure the function of intracellular bacteria within eukaryotic cells. This is shown by the quantitative imaging of the incorporation of ^15^N in *T. turnerae *living in the gill bacteriocytes of a shipworm (*Lyrodus pedicellatus*) raised under a ^15^N atmosphere, as shown in the HSI image of the ^12^C^15^N^-^/^12^C^14^N^- ^ratio (Figure 5g). The bacteria in the bacteriocytes that have incorporated ^15^N are shown in colors between yellow and magenta (the shipworm tissue is blue). An internal control is the isotope ratio ^13^C^-^/^12^C^- ^of the same field (Figure 5h), which shows a lack of contrast. The uniformity of the carbon ratio image eliminates the possibility of artifacts in the nitrogen ratio image as a result of morphologically or instrumentally induced isotope fractionation.

These quantitative images show that the MIMS method will be a powerful tool in the investigation of nitrogen fixation. It can also be used to study bacteria in natural environments and to explore the activity of diazotrophic symbionts in the tissues of plants and animals. It is worth noting that the size of an object can be estimated directly from the pixel signal intensity. This is illustrated with the flagellum visible on one *T. turnerae *cell (Figure 5i). For example, at mass ^12^C^15^N (Figure 5j), we have a mean of 1,473 counts per pixel on the bacterium (Figure 5k, inset, red box). A line profile of the counts per pixel across the flagellum of *T. turnerae *at mass ^12^C^15^N is shown in Figure 5k. The pixel crossed by the flagellum registered 66 counts. All conditions being approximately the same, the number of counts is proportional to the surface area of the material sampled in one pixel. In this particular image of 60 × 60 μm, 256 × 256 pixels, one pixel is equivalent to 234 nm covering an area of 54,756 nm^2^. If the length of the flagellum crosses a pixel, 66 counts would represent a width of (54,756 nm^2^/1,473 counts) × (66 counts/234 nm) = 10.5 nm; using the count values for the same pixels at mass ^12^C^14^N, the estimate is 10.6 nm, which is approximately the diameter of a *T. turnerae *flagellum.

#### Use of double labeling with bromodeoxyuridine and ^15^N-leucine to measure protein renewal

Because MIMS analysis sputters only a few atomic layers, a sample can be reanalyzed many times. This is illustrated by double-labeling studies of protein renewal and DNA replication in the mouse kidney after ischemia. We have previously shown [32] in the mouse that 30 minutes of bilateral renal ischemia, resulting in significant increases of blood urea nitrogen and creatinine, leads to protection of the mouse kidney against a subsequent ischemic insult 8 or 15 days later, even when the second ischemic period is extended to 35 minutes. Graded levels of time of initial ischemia resulted in graded levels of protection 8 days later. Bromodeoxyuridine (BrdU) and ^15^N-leucine were administered to mice subsequent to the first ischemia, in order to characterize the different response in cell proliferation in preconditioned and non-preconditioned kidneys exposed to ischemia on day 8 after the initial surgery. Quantitative MIMS images were recorded from thin sections of epon-embedded kidneys. The images were acquired in parallel at mass ^12^C^14^N to show a morphological overview, at mass ^31^P to view the cell nuclei and at mass ^81^Br to identify the nuclei undergoing DNA replication, as shown by BrdU incorporation. Protein renewal was calculated from parallel imaging at mass ^12^C^14^N and mass ^12^C^15^N.

**Figure 6:**
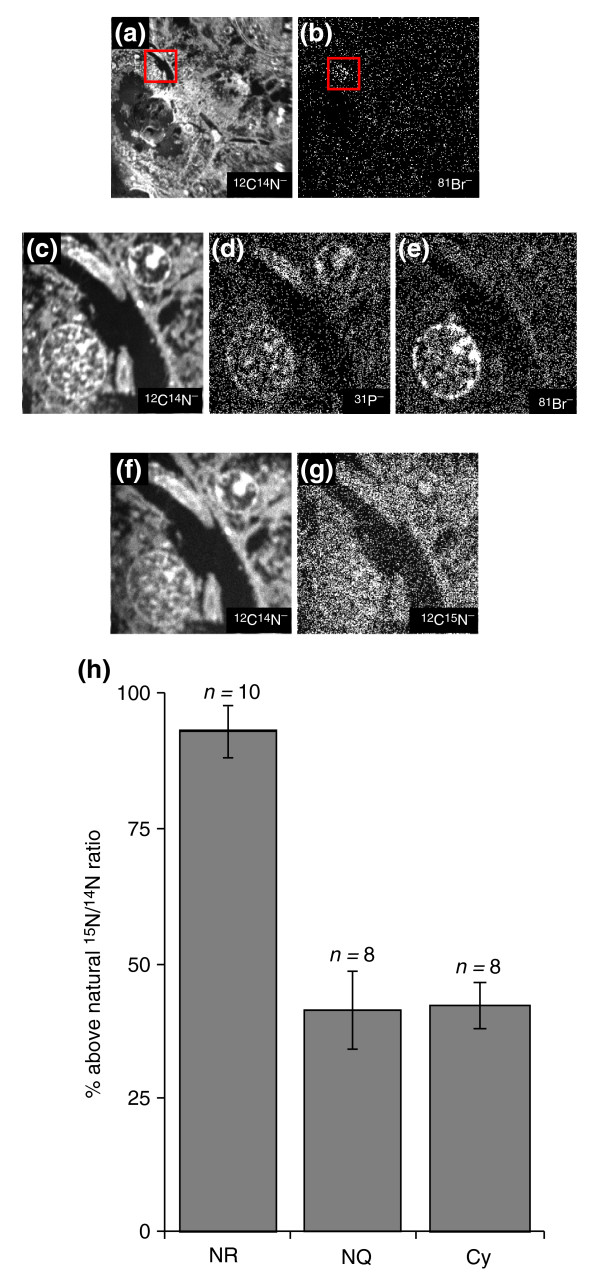
Cell replication and protein renewal in post-ischemic mouse kidney analyzed with double labeling with BrdU, analyzed as ^81^Br- and ^15^N-leucine. **(a,b) **Wide-view parallel quantitative mass image of (a) ^12^C^14^N^- ^and (b) ^81^Br^-^. The ^81^Br^- ^label indicates a cell with replicated DNA. Field: 100 μm × 100 μm (256 × 256 pixels); acquisition time 2 min. **(c-e) **Higher-resolution parallel images of the boxed regions in (a,b) for (c) ^12^C^14^N^-^; (d) ^31^P^-^; (e) ^81^Br^-^. The ^31^P^- ^image enables identification of other cells with unreplicated DNA. Field: 23 × 23 μm (256 × 256 pixels); acquisition time 60 min. **(f,g) **Parallel quantitative mass images for (f) ^12^C^14^N^- ^and (g) ^12^C^15^N^-^, from which protein renewal is calculated. Field: 23 × 23 μm (256 × 256 pixels); acquisition time 10 min. **(h) **Quantitation of protein renewal in replicating and non-replicating cells. Cy, cytoplasm; NQ, nucleus of non-replicating cells; NR, nucleus of replicating cells.

A first MIMS analysis of a 100 × 100 μm field for 2 minutes, as shown in Figure 6a,b, reveals that one nucleus (in the ^81^Br^- ^image in Figure 6b) has replicated its DNA, whereas the others have not. A second MIMS analysis at higher resolution of cells around this replicating nucleus is shown in Figure 6c–e. The replicating nucleus is seen in the ^81^Br^- ^mass image (Figure 6e), and the ^31^P^- ^image (Figure 6d) shows the presence of two other nuclei that did not replicate. A third MIMS analysis was performed on the same field at masses ^12^C^14^N (Figure 6f) and ^12^C^15^N (Figure 6g) to quantitate the protein renewal and compare renewal between replicating and non-replicating cells. We found that incorporation of ^15^N into the replicating nuclei was twice as high as that in either the cytoplasm or in non-replicating cells (nuclei or cytoplasm; Figure 6h). The ^12^C^14^N images acquired successively (Figure 6c,f) show that there are no visible changes; this validates the comparison of data obtained in the second and third analysis. MIMS can thus be used with multiple tags that can be studied with a succession of parallel analyses of the same field at a variety of isotope combinations.

### Qualitative labeling with stable isotopes

**Figure 7:**
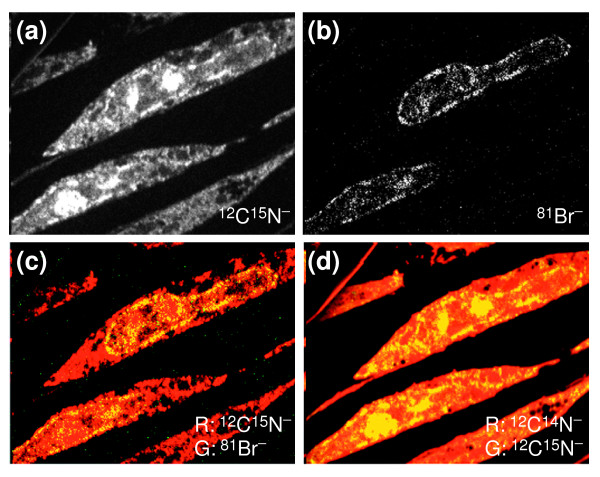
Qualitative co-localization of DNA and RNA through simultaneous imaging of RNA and DNA. Rat embryo fibroblasts were pulsed with ^15^N-uridine and BrdU as markers of newly synthesized RNA and DNA, respectively. **(a,b) **Parallel mass images at (a) ^12^C^15^N^- ^and (b) ^81^Br^-^. **(c) **Overlay of ^12^C^15^N^- ^and ^81^Br^- ^images. ^12^C^15^N^- ^is depicted as red (R) and ^81^Br^- ^as green (G); the overlap between them shows up as yellow. **(d) **Overlay of ^12^C^14^N^- ^and ^12^C^15^N^- ^images. ^12^C^14^N^- ^is depicted as red (R) and ^12^C^15^N^- ^as green (G); the overlap between them shows up as yellow. Conditions of MIMS analysis: beam current 2pA; beam diameter 100 nm; field 20 × 20 μm.

MIMS methodology enables us to study the spatial aspects of metabolic pathways and the spatial relationship between replication and transcription. As an example, we will show MIMS atomic mass imaging of the co-localization of RNA and DNA. Rat embryo fibroblasts were pulsed with ^15^N-uridine and BrdU, markers of newly synthesized RNA and DNA, respectively. The simultaneously recorded distributions of ^12^C^15^N^- ^and ^81^Br^- ^are shown in Figure 7a,b. As expected, the bromine signal (DNA) is restricted to the cell nuclei; there is strong ^81^Br labeling along the nuclear envelope and around the nucleoli (Figure 7b). In contrast, the ^12^C^15^N^- ^signal (RNA) is strong within the nucleoli and along the nuclear envelope (Figure 7a).

An overlay of the ^12^C^15^N^- ^signal in red with the ^81^Br^- ^signal in green shows the co-localization of newly synthesized RNA and DNA in yellow (Figure 7c). This co-localization, visualized directly from the isotope images, avoids the complications potentially introduced by immunochemical methods [33]. Localization of newly synthesized RNA requires distinguishing a local excess of ^15^N over its natural occurrence. We carried out another MIMS analysis of the same cells to record the distributions of ^12^C^14^N^- ^and ^12^C^15^N^- ^in parallel (in our prototype instrument, we cannot simultaneously detect the isobars ^12^C^14^N^- ^and ^12^C^15^N^- ^together with ^81^Br^- ^because of the steric hindrance of the electron multipliers). Overlaying the ^12^C^14^N^- ^signal in red with the ^12^C^15^N^- ^signal in green shows up the local excesses of ^15^N above its natural occurrence in yellow (Figure 7d). The yellow identifies the localization of ^15^N-uridine-labeled newly synthesized RNA within the nucleoli, along the nuclear envelope, and in the cytoplasm of the top cell.

**Figure 8:**
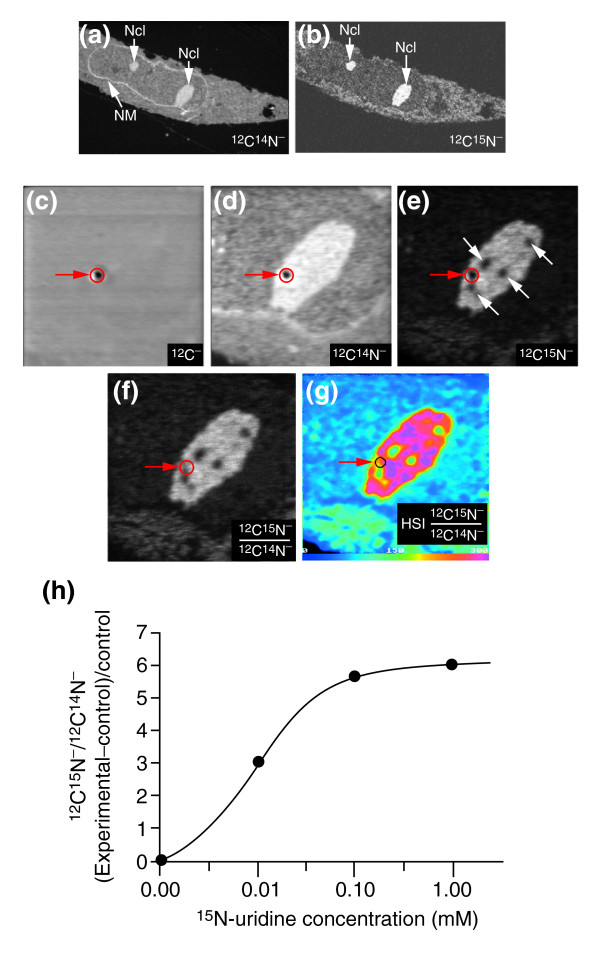
Distinguishing between an artifact and the subnucleolar heterogeneity of ^15^N-uridine incorporation. **(a,b) **Parallel quantitative mass images of (a) ^12^C^14^N^- ^and (b) ^12^C^15^N^- ^images of a fibroblast cultured in the presence of ^15^N-uridine. Ncl, nucleoli; NM, nuclear membrane. Field: 40 × 40 μm (image has been cropped); acquisition time 20 min. **(c-e) **High-resolution parallel mass images at ^12^C^-^, ^12^C^14^N^- ^and ^12^C^15^N^- ^of the large nucleolus seen in (a,b). Field: 8 × 8 μm; acquisition time 30 min. (c) ^12^C^- ^image, arising from both tissue and embedding medium; the dark spot (red arrow) was caused by accidental exposure to a stationary high-intensity primary Cs^+ ^ion beam. (d) ^12^C^14^N^- ^image. (e) ^12^C^15^N^- ^image, showing subnucleolar areas of low local ^15^N incorporation (white arrows). **(f) **Ratio of the (d) ^12^C^14^N^- ^and (e) ^12^C^15^N^- ^images; here, the 'dark spot' (red circle) is barely visible because the value of the ^12^C^15^N^-^/^12^C^15^N^- ^ratio is close to that of the surrounding area. **(g) **HSI image of the ^12^C^15^N^-^/^12^C^14^N^- ^ratio (the numerator has been multiplied by 10,000). The 'dark spot' isotope ratio is close to that of the surrounding area. Subnucleolar regions of low incorporation of ^15^N-uridine stand out in both the (f) ratio and the (g) HSI images. **(h) **Calibration with ^15^N-uridine. The graph shows the intranucleolar accumulation of ^15^N-uridine (measured as ^12^C^15^N^-^/^12^C^14^N^- ^(experimental – control)/control) as a function of the concentration of ^15^N-uridine in the culture medium.

The importance of parallel detection and isotope ratio imaging is illustrated by the following fortuitous observation. We used MIMS to study the distribution of RNA in the nucleolus by studying fibroblasts cultured in the presence of ^15 ^N-uridine. Quantitative mass images of ^12^C^-^, ^12^C^14^N^- ^and ^12^C^15^N^- ^secondary ions were acquired in parallel. In the fibroblast shown in Figure 8, the nuclear membrane is clearly visible at mass ^12^C^14^N^- ^(Figure 8a), and two nucleoli are seen highly contrasted at masses ^12^C^14^N^- ^and ^12^C^15^N^- ^(Figure 8a,b). Higher resolution parallel mass images of the nucleolus seen on the right in Figure 8a are shown in Figure 8c–e. In these cells embedded in epon – a polymer lacking nitrogen – the ^12^C^- ^image (Figure 8c) shows little contrast except for a dark spot (diameter around 122 nm) in the middle (Figure 8c, red arrow; the low brightness indicates low counts). This spot was caused by accidental exposure of the sample to an intense stationary primary-ion beam. The spot is also seen in the ^12^C^14^N^- ^and the ^12^C^15^N^- ^images (Figure 8d,e). The ^12^C^15^N^- ^image, however, contains four additional dark regions (Figure 8e, white arrows), ranging from 200 to 280 nm in diameter. Nevertheless, isotope ratio and HSI derivation of ^12^C^15^N^- ^and ^12^C^14^N^- ^images clearly distinguish the accidental dark spot, which has a high level of ^15^N incorporation, from the other four sub-nucleolar regions, which have low ^15^N incorporation; they can therefore be taken to be related to nucleolar organization (Figure 8f,g). We also used MIMS to evaluate the dose response of uridine incorporation in nucleoli of rat embryo fibroblasts cultured in the presence of 0.0, 0.01, 0.1, and 1.0 mM ^15^N-uridine (Figure 8h), demonstrating that MIMS may be used to establish a dose-response curve at the level of intracellular organelles.

### Use of MIMS without isotope labeling to study gross differences in subcellular composition

**Figure 9:**
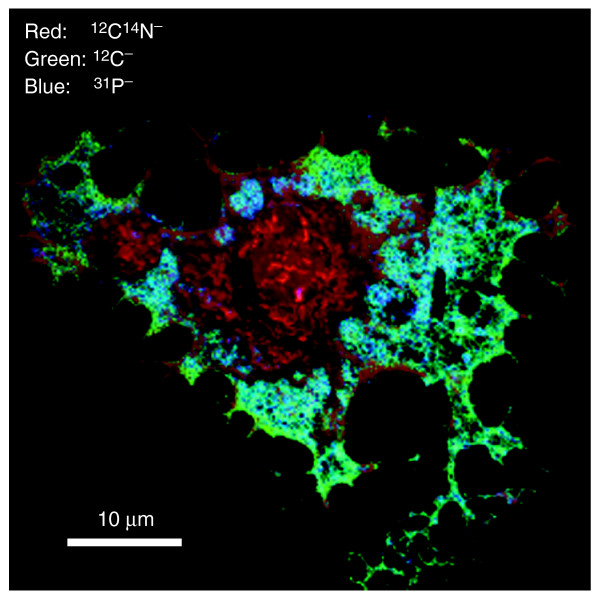
Analysis of gross differences in composition within an unlabeled cell. Endothelial cells were cultured on silicon supports, fixed on the support, dried, and analyzed with MIMS. Quantitative mass images of the surface of a whole endothelial cell were recorded in parallel at masses ^12^C^-^, ^12^C^14^N^- ^and ^31^P^-^. An overlay of these images is shown, with ^12^C^14^N in red, ^12^C in green, and ^31^P in blue. Scale bar = 10 μm.

Quantitative mass images of the chemical elements within a cell can provide information on the existence and location of subcellular domains with gross differences in composition. Thus, even without exposing the cells or tissues to isotopically labeled molecules, we may obtain a measure of the gross cellular composition at the level of microdomains that cover areas of sub-micrometer size. This is illustrated by the overlay image of an endothelial cell analyzed in parallel for ^12^C^-^, ^12^C^14^N^- ^and ^31^P^- ^(Figure 9). Striking differences in gross composition within a cell are revealed. The area over the nucleus and the thicker part of the cytoplasm is intensely red, an indication of comparatively high nitrogen content; the wide area at the periphery (the lamellipodium) is relatively rich in phosphorus and poor in nitrogen; and at the outmost edge of the cell, there is relatively more carbon than in the wide part of the lamellipodium. Short, thin protrusions with a relatively high nitrogen signal can also be seen at the very edge of the cells; these are probably filopodia. One may assume the following: high ^12^C^14^N indicates protein (or glycoproteins); high ^12^C^14^N associated with phosphorus indicates nucleotides; ^12^C with less ^12^C^14^N indicates lipids or sugars; and ^12^C associated with ^31^P indicates phospholipids.

**Figure 10:**
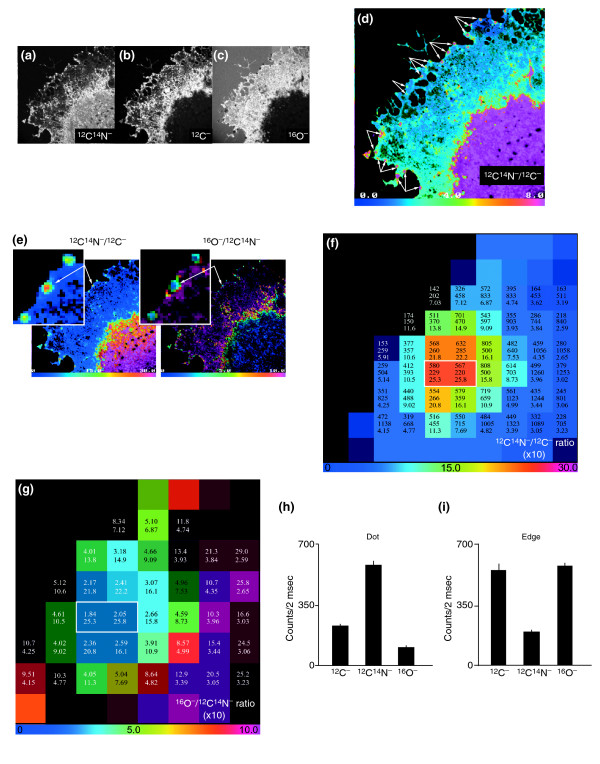
A detailed analysis of the edge of a lamellipodium of an unlabeled endothelial cell. This example illustrates analysis by counts per pixel and HSI. **(a-c) **Three MIMS images acquired in parallel at (a) ^12^C^14^N^-^, (b) ^12^C^-^, and (c) ^16^O^-^. Field: 60 × 60 μm (256 × 256 pixels); acquisition time 2 min. **(d) **HSI image of the ratio ^12^C^14^N^-^/^12^C^- ^(the numerator has been multiplied by 10). Magenta dots (arrowed) indicating areas of high relative ^15^N incorporation appear at the edge of the lamellipodium. Field: 60 × 60 μm (256 × 256 pixels). **(e) **HSI images of the ratios ^12^C^14^N^-^/^12^C^- ^(left) and ^16^O/^12^C^14^N^- ^(right; the numerator has been multiplied by 10). The regularly spaced dots (arrowed) can be seen at the edge of the lamellipodium. **(f) **At each pixel, arranged from top to bottom, are the values of the ^12^C^14^N^- ^counts, the ^12^C^- ^counts and the ^12^C^14^N^-^/^12^C^- ^ratio (multiplied by 10) for the pixels shown in the inset in (e). **(g) **The corresponding values at each pixel, arranged from top to bottom, for the ^12^C^14^N^-^/^12^C^- ^ratio values (multiplied by 10) and the ^16^O^-^/^12^C^14^N^- ^ratio (multiplied by 10). **(h,i) **Bar graph of the mean count values of ^12^C^-^, ^12^C^14^N^- ^and ^16^O^- ^on (h) the dot at the periphery of the lamellipodia and (i) the edge of the lamellipodia.

We undertook a more detailed analysis of the region at the edge of the lamellipodia of endothelial cells (see Figure 9), in which we saw domains two pixels wide (the pixel size here is 234 nm) containing five times more nitrogen and a tenth the level of oxygen than the neighboring pixels. From the original MIMS images acquired in parallel at masses ^12^C^14^N^-^, ^12^C^- ^and ^16^O^- ^(Figure 10a–c), together with HSI images of the ratio ^12^C^14^N^-^/^12^C^- ^(Figure 10d) and of the ratio ^16^O^-^/^12^C^14^N^- ^(not shown), we observe lamellipodial domains at the edge of the cell that look like regularly spaced 'dots'. These are rich in ^12^C^14^N and poor in ^16^O compared with their surroundings. The values of ^12^C^14^N^- ^counts, ^12^C^- ^counts and of the ^12^C^14^N^-^/^12^C^- ^ratio are shown in Figure 10f for a group of pixels constituting the central dot of the inset in Figure 10e, and the values of the ^16^O^-^/^12^C^14^N^- ^and of the ^12^C^14^N^-^/^12^C^- ^ratios are shown in Figure 10g for the same pixels; these panels illustrate the way in which quantitation and imaging are intimately associated in MIMS. The white rectangle in Figure 10f,g surrounds two neighboring pixels that have the highest nitrogen content and the lowest oxygen content (Figure 10h) compared with the surrounding lamellipodium (Figure 10i). Thus, parallel quantitative mass imaging using MIMS without isotopic supplementation can identify nanometer-sized structures that may be functionally significant.

### Labeling with radioactive ^14^C

**Figure 11:**
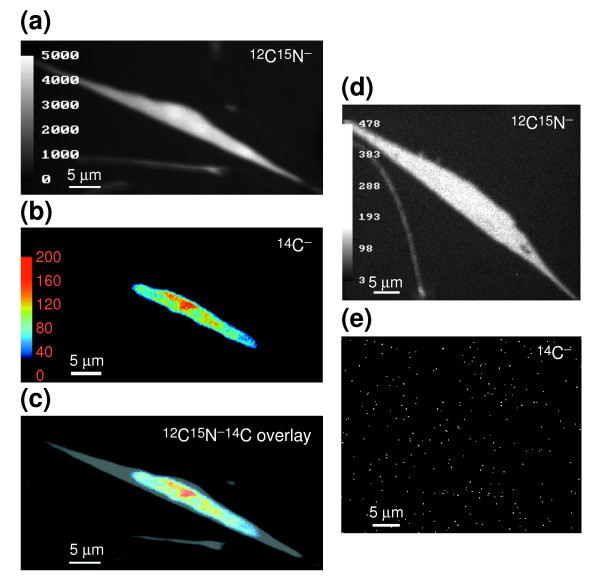
Rat embryo fibroblasts labeled with ^14^C-thymidine. Fibroblasts were cultured on silicon chips, deprived of serum for 24 h and pulsed with serum and 19 nmol ^14^C-thymidine/ml (1 mCi/ml). **(a,b,c) **Simultaneous quantitative mass images of a fibroblast at (a) ^12^C^15^N^- ^(grayscale); (b) ^14^C^- ^(pseudo-color); (c) overlay of the ^14^C^- ^and ^12^C^15^N^- ^images. Field: 50 × 26 μm; acquisition time 14 h. **(d,e) **Simultaneous quantitative mass images of a control rat embryo fibroblast at (a) ^12^C^15^N^-^; (b) ^14^C^-^. Field: 50 × 41 μm; acquisition time 2 h.

MIMS opens the world of stable isotopes to quantitative nanoautography. It is similar in principle but much more powerful than autoradiography because it is precisely quantitative, needs much shorter exposure times, can use the very large number of stable isotopes that are naturally available, can easily use multiple labels, gives high lateral resolutions, provides exceptional depth resolution and would be harmless for clinical use. MIMS can also be used for high-resolution quantitative imaging of radioisotopes (such as ^14^C), and with high sensitivity, as shown in the pioneering work of Hindie *et al*. [10]. An example of parallel imaging at masses ^14^C^- ^and ^12^C^15^N^- ^of a whole fibroblast pulsed with serum and then with ^14^C-thymidine after serum deprivation is shown in Figure 11a,b, and the overlay of the ^12^C^15^N^- ^and ^14^C^- ^images in Figure 11c. The ^12^C^15^N^- ^and ^14^C^- ^images from a control sample with no added ^14^C are shown in Figure 11d,e. The ^12^C^15^N^- ^mass image maps the whole fibroblast (Figure 11a). The ^14^C^- ^atomic mass image, indicative of the ^14^C-thymidine in the DNA, is restricted to the nucleus (mean count within nucleus = 49.0, SD = 38.2, *n *pixels = 2,388, sum of counts = 116,955). The ^14^C signal is variable across the nucleus and is segregated into domains, reminiscent of the results of Wei *et al*. [34]. The mean background ^14^C count outside the fibroblast is 0.6 (SD = 1.1, *n *pixels = 31,148, sum of counts = 19,873). The mean ^14^C count in the nuclear region of the control sample is 0.009 (SD = 0.128, *n *pixels = 2,875, sum of counts = 25). The mean ^14^C count outside the nucleus of the control sample is not significantly different, with a value of 0.008 (SD = 0.153, *n *pixels = 50,842, sum of counts = 420). Thus after exposure to 19 nmol/ml of ^14^C-thymidine, counts in the nucleus of the labeled sample have increased by a factor of 6,125 relative to the control sample. Control background count of ^14^C is negligible. Compared with autoradiography, one can estimate that even if only 1‰ of the ^14^C atoms were sputtered and only 1% of the sputtered atoms were ionized, the sensitivity of MIMS is at least 10^3^-fold greater (see Additional data file 5 for calculations).

### Use of MIMS to track individual cells in large cell populations

Following the transplantation of allogeneic tissues and organs, some donor-derived cells are known to leave the graft and present alloantigens to the host's immune system [35-37]. The exact nature and frequency of the donor cells infiltrating the host's lymphoid tissues are still unclear, however. The movement patterns of recipient immuno-competent cells that recognize the alloantigens are also unknown. These questions have remained unanswered because of the lack of sufficiently sensitive techniques for accurately tracking and visualizing small numbers of lymphocytes in tissues. Here, we use MIMS to track donor cells after transplantation.

**Figure 12:**
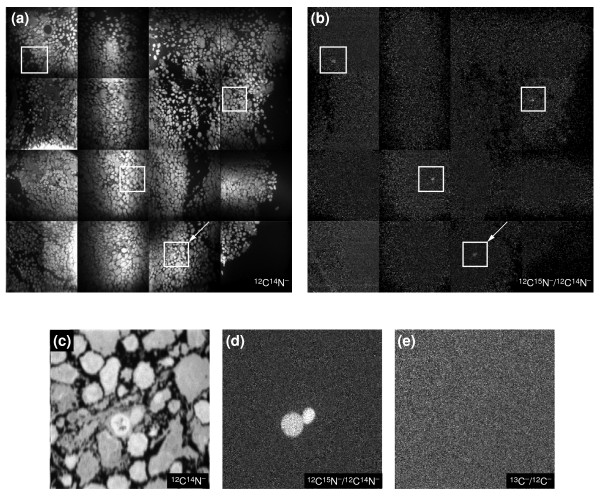
Tracking donor cells in a popliteal lymph node of a BALB/c mouse that has been injected in the footpad with 2 × 10^7 ^spleen cells from a C57Bl/6 mouse fed for 2 weeks on a ^15^N diet. **(a,b) **Parallel quantitative mass imaging at ^12^C^14^N^- ^and ^12^C^15^N^-^. (a) A mosaic of ^12^C^14^N^- ^images, showing the topology of a lymph-node section. (b) A mosaic of the ^12^C^15^N^-^/^12^C^14^N^- ^ratio images, showing ^15^N-labeled donor cells. Tile field: 100 μm × 100 μm (256 × 256 pixels); acquisition time 2 min per tile (16-tile mosaic). **(c-e) **Higher-resolution parallel mass imaging at ^12^C^-^,^13^C^-^, ^12^C^14^N^- ^and ^12^C^15^N^- ^of the field indicated by the arrow in (a,b). (c) ^12^C^14^N^- ^image, (d) ^12^C^15^N^-^/^12^C^14^N^- ^ratio image and (e) ^13^C^-^/^12^C^- ^ratio image of the same field. Field: 30 × 30 μm (256 × 256 pixels); acquisition time 40 min.

Spleen cells from C57Bl/6 (B6, MHC haplotype H-2^b^) mice fed for 2 weeks with a ^15^N diet were injected subcutaneously in the footpad of a fully allogeneic BALB/c mouse (H-2^d^). Two days later, popliteal lymph nodes (located behind the knee, where they drain the foot pad of the mouse) were collected and examined by MIMS for the presence of donor cells. Mass images of the ^12^C^-^, ^13^C^-^, ^12^C^14^N^- ^and ^12^C^15^N^- ^ions were acquired in parallel. Because only a very few donor cells are expected to be found among the recipient cells, a large number of cells need to be examined relatively quickly with some means of highlighting the areas that could contain donor cell(s). This was done using MIMS to rapidly map a large area of lymph node as shown in Figure 12a,b. We clearly observe areas where there appears to be a small excess of ^15^N above its natural abundance (Figure 12b). These areas were then reanalyzed with MIMS at a higher resolution over a longer time. A field containing donor cells is shown in Figure 12c–e. In the ^12^C^15^N^-^/^12^C^14^N^- ^ratio image (Figure 12d), two donor cells are clearly visible. The ratio image of ^13^C^-^/^12^C^- ^of the same field taken simultaneously (Figure 12e) does not show any contrast and acts as an internal control. The frequency of transplanted cells infiltrating the recipient lymph node was estimated to be 690 cells per million.

We conclude that the MIMS technique allows the detection and frequency determination of rare cells infiltrating the recipient's lymphoid tissues after transplantation. The methodology can be extended to study solid organ transplants and the nesting of stem cells. It is important to note that the current techniques for tracking transplanted cells are fraught with difficulties [38]. Labeling DNA with stable isotopes should provide a method allowing long-term and physiological marking, the possibility of determining the generation of single cells from the amount of label within the cell, and the possibility of labeling both donor and recipient cells differently, enabling the unambiguous recognition of cell fusion or phagocytosis of donor cells by the recipient's cells [38].

## Discussion

We have shown that we can obtain quantitative atomic mass images using MIMS. Because these images are produced in parallel from the same sputtered volume they are in exact register with each other, which is necessary to derive meaningful isotope ratios. A lateral resolution that reaches 33 nm, in theory, and a high mass resolution (a mass/change in mass ratio of approximately 10,000) at high secondary-ion transmission (70–80%) allows us to image and quantitate molecules labeled with stable (or radioactive) isotopes within subcellular compartments. The high mass resolution allows us to distinguish the distribution of isobaric atomic species. For example, by separating ^12^C^15^N^- ^from ^13^C^14^N^- ^and ^13^C^- ^from ^12^C^1^H^-^, we can image the intracellular distribution of molecules labeled with the stable isotopes ^13^C and ^15^N (such as ^15^N-thymidine and ^13 ^C-uridine) in parallel and in the same preparation. Finally, the high stability of the primary beam, the mass spectrometer and the detectors contribute to precise quantification. These unique characteristics have allowed us to develop MIMS.

### Instrumental advances

#### Instrument modifications

**Figure 13:**
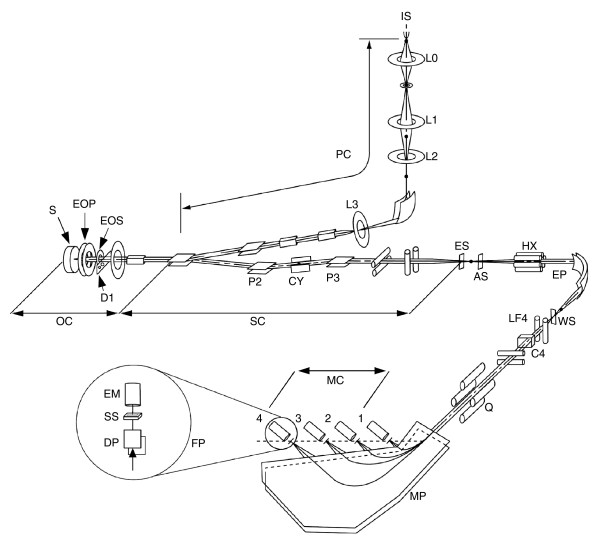
Diagram of the prototype NanoSims50 used in MIMS. The main components of the instrument include: the primary column (PC) with a cesium primary-ion source (IS) used to sputter the surface of the sample and to enhance the yield of negative secondary ions and a series of lenses (L0, L1, L2 and L3) to shape the primary beam; the objective column (OC), where the same ion optic is used in a coaxial manner both to focus the primary beam on the sample (S) and to collect the secondary ions (the primary-ion beam is focused on the sample with the objective lens (EOP) and aperture limited with the diaphragm (D1), the secondary ions are collected with the secondary-ion focusing lens (EOS)); the secondary column (SC) where the secondary-ion beam is shaped to match the acceptance of the spectrometer (the secondary column contains an entrance slit (ES), a corrector (CY) and deflectors (P2 and P3) to center the secondary-ion beam in the entrance slit); an aperture slit (AS) to reduce the angular aberration of the secondary-ion beam; and the mass spectrometer, made up of the association of an electrostatic prism (EP) and a magnetic prism (MP), which enables the focusing of the secondary ions as narrow lines along the focal plane (FP) of the magnet. Chromatic aberrations are minimized with the quadrupole (Q) and the slit lens (LF4). Energy aberration is reduced with the energy slit (WS). The transmission at high mass resolution is improved by correcting the main second-order aperture aberrations with the hexapole (HX) placed in front of the electrostatic prism, tilting the entry and exit faces of the magnetic prism, and focusing in the vertical section using additional lenses placed between the electrostatic and magnetic prisms. The deflectors (C4) help to center the masses in the detectors. In the multi-collection chamber (MC), four detectors can be moved along the focal plane. Each detector is made up of deflection plates (DP) followed by a selection slit (SS) and a miniature electron multiplier (EM). The deflection plates, which permit scanning of a small portion of the spectrum, greatly improve the final tuning of mass lines. This instrument provides both parallel detection and high mass resolution with little sacrifice in secondary-ion transmission from sample to detector. As a particularly striking example, in Figure 15, the ^12^C^- ^secondary ions were detected at 90% relative transmission with a mass resolution of 1 part in 11,825.

In SIMS, a primary-ion beam (usually Cs^+ ^or O^-^) is used as a probe to scan across the surface of a solid sample. The impact of a primary ion on the surface of the sample triggers a cascade of atomic collisions, and atoms and clusters of atoms are ejected, most originating in the neighborhood of the impact point. In the ejection process, some of them will be spontaneously ionized; these secondary ions are characteristic of the composition of the target area (see Figure 1). After separating the secondary ions according to their masses, an image of the surface composition in respect of a selected mass can be recorded. We used a new generation of SIMS instrument that can detect several secondary ions at once; this and several other advances detailed below represent major improvements over previous generations of SIMS. The machine is the prototype of the NanoSims50 built at ONERA (Office National d'Études et de Recherches Aérospatiales) jointly with UPS (Université Paris-Sud) and upgraded by Cameca, Courbevoie, France, and is shown in Figure 13.

The objective column (OC) in the new SIMS instrument (see Figure 13), where the primary ions and secondary ions share the same path but in opposite directions, is a big step forward. It allows for a very short focal length for the ion-beam focusing lens (EOP) and for the secondary ion extracting lens (EOS) leading to a significant reduction in various aberrations. In addition, the incidence of the coaxial primary-ion and secondary-ion beams normal to each other allows us to reduce the geometrical dimensions of the instrument, leading to a significant reduction in various aberrations. The improvement rests on using the same lens for focusing the primary-ion beam, and for collecting the secondary ions that are formed. Secondary ions are emitted at a wide range of angles and energies. To collect them efficiently a strong electrostatic accelerating field is needed near the sample surface to gather their trajectories close to the surface normal. The conjugate action of the immersion lens and other lenses in the secondary optical column gives the secondary ion beam springing from the primary ion probe impact area the appropriate shape for matching the acceptance of the mass spectrometer tuned for high mass resolution. It should be emphasized that getting high mass resolution and high collection efficiency together is possible because the impact area of the primary-ion beam is very small (less than 0.1 μm^2^). This allows us to keep the beam small during its transport from the sample to the entrance of the spectrometer. From the general laws of optics, however, we can do this only for one position of the beam on the surface. As soon as the primary-ion beam moves, the secondary-ion beam emitted from the impact area is displaced as a whole in front of the spectrometer and a variable fraction of the beam (or no beam at all), depending upon the amplitude of the motion, enters the spectrometer.

To solve this problem, the machine uses an operation mode called 'dynamic transfer' to keep the secondary-ion beam stationary at the entrance of the spectrometer while the probe scans the surface. Dynamic transfer is achieved by the set of deviation plates, which move in synchrony with the scanning primary-ion beam. Because of the coaxial layout, those plates move both secondary and primary beams, but whereas they act on the secondary beam to align it along the optical axis, they act on the primary beam to make it rotate about the center of the aperture stop (see D1, Figure 13), which limits aperture aberrations on the probe. As a result, not only does the secondary beam stay stationary, which ensures a constant collection efficiency over the scanned area, but the intensity of the primary beam remains constant during the scanning; both of these avoid the 'vignetting', which is a gradual darkening of the corners of the ion images as the size of the field of view increases.

High mass resolution is obtained because the initial energy of ion spread has been cancelled at the level of the mass lines by coupling the electrostatic and magnetic prisms via a quadrupole lens. In addition, the elaborate ion optics allows the substantial reduction of second-order aperture aberrations, which improves the mass-resolving power (for example, at mass 27 the separation of ^12^C^15^N from ^13^C^14^N). This results from both the suppression of second-order aperture aberrations of the magnetic prism by tilting the entrance face of the magnetic prism, and the reduction of second-order aberrations of the electrostatic prism by placing a hexapole in front of the electrostatic prism (see Figure 13).

The mass spectrum of the secondary ions is displayed along the focal plane as a set of well-resolved mass lines. It is a discrete spectrum with wider spacing between ions separated by 1 mass unit and closer spacing when multiplets (isobaric species) occur. Because of the limited size of the magnet, however, the whole spectrum cannot be displayed at once. The ratio between the lower and the higher masses in the displayed range is about 20. By changing the strength of the magnetic field it is possible to choose the lower mass and, in consequence, the higher one. For instance, if 12 is the lower mass unit, the spectrum is displayed up to mass number 240.

**Figure 14:**
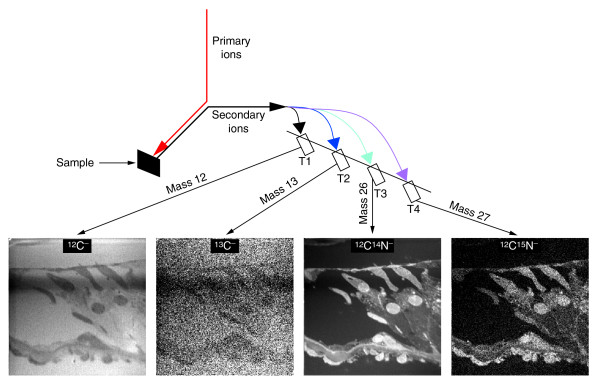
Parallel quantitative mass imaging with MIMS, using a cochlear field as an example. A schematic representation of the MIMS instrument is shown at the top, with the secondary ions beings selected in parallel by four detectors (T1–T4) which are moved along the focal plane to the appropriate positions for a given mass. The ^12^C^-^, ^13^C^-^, ^12^C^14^N^- ^and ^12^C^15^N^- ^quantitative mass images recorded by the four detectors are shown below.

Mass selection for discriminating the secondary ions is provided by individual detector 'trolleys' in the mass spectrometer, each containing a set of deflection plates followed by a selection slit and an electron multiplier (see Figure 13), which are moved along the focal plane to the appropriate positions corresponding to the masses of interest. The positions depend on the kinetic energy of the secondary ions and the magnetic field strength of the mass spectrometer. For example, in the analytical run shown in Figure 14, one trolley was positioned for detecting atomic mass 12, a second for mass 13, a third for mass 26, and a fourth for mass 27. As stated earlier, the ability to detect several masses simultaneously at high mass resolution is novel, and is essential to derive meaningful isotope ratios from a sub-cubic-micrometer sample volume. Thus, in the example shown in Figure 14, it is necessary to distinguish at mass number 27 between ^12^C^15^N^- ^and ^13^C^14^N^-^. This last level of mass selection is conveniently obtained by operating the deflection plates in front of the selection slit.

**Figure 15:**
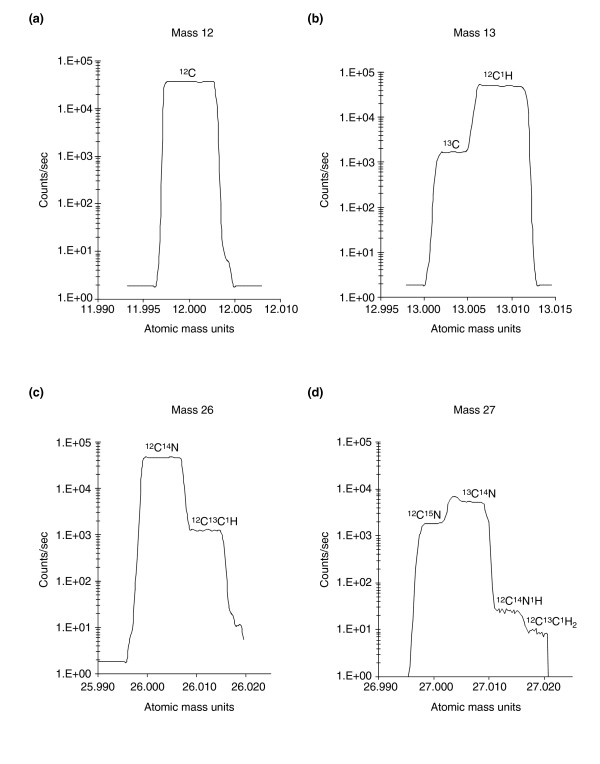
High-resolution mass spectra illustrate the resolving power of our prototype NanoSims instrument. The detectors are positioned along the focal plane at the focal points for the atomic masses **(a) **12, **(b) **13, **(c) **26, and **(d) **27 as shown in Figure 14. Varying the electrical potential of the deflector plates selects between different isobaric ions. Note that with the log scale and the counting software, when zero counts are recorded, the data point is recorded as 1/*T*, where *T *is the counting time in seconds for each data point. In (a-c) the counting time was 0.5 sec, and the background count rate appears as 2 counts/sec even though it is zero counts; in (d), the counting time was 10 sec and a background count rate of zero is not visible. See Additional data file 6 for more details on the shape of the peaks.

Recordings from individual electron multipliers when the deflection plate voltages are adjusted gradually so as to scan across a narrow range of secondary-ion masses are shown in Figure 15. By setting the voltages appropriately it is possible to scan finely the multiplet of the already highly resolved mass lines and to determine which voltage is suitable for isolating the mass of interest, practically eliminating any spillover from neighboring mass peaks. It should be noted that the width of the mass selection slit is larger than the mass line itself. This configuration allows us to make precise isotopic measurements because it allows for small drifts of the mass line that can result from uncontrolled instrumental factors. As a consequence, the mass line appears in the recordings as a peak with a flat top (Figure 15). Two adjacent mass lines can be completely separated and appear in the recording with no valley between them (Figure 15b–d and Additional data file 6). It can be seen from Figure 15b–d that different isobars with very similar masses can be distinguished, such as ^13^C^- ^and ^12^C^1^H^- ^(mass 13.003355 and mass 13.007825, respectively; Figure 15b); ^12^C^14^N^- ^and ^1^H^12^C^13^C^- ^(mass 26.003074 and mass 26.011180, respectively; Figure 15c); ^12^C^15^N^-^, ^13^C^14^N^- ^and ^12^C^14^N^1^H^- ^(mass 27.000109, mass 27.006429 and mass 27.010899, respectively; Figure 15d).

A single ion impact on the first dynode of an electron multiplier ejects several secondary electrons, which are subsequently amplified by the other dynodes in a chain amplification process and generate a voltage pulse. Pulse heights depend upon the ion-electron conversion yield on the first dynode and upon the voltages applied on other dynodes. Pulse heights have a distribution that can be stretched by changing the high voltage. The distribution is usually depleted on the low-amplitude side, where one must account for the superposition with low-amplitude pulses from the electronic noise. A pulse-height discriminator eliminates low-amplitude contributions so as to select only signal pulses. Of course, a fraction of the signal is suppressed in that process, but this fraction is quite small, for example a few percent for CN^-^. The secondary-ion detection efficiency is set to be equivalent among the detectors in part by adjusting the rejection threshold and in part by adjusting the high voltage. Measurement of samples of known isotopic composition can be used to correct for any remaining differences in efficiency between detectors. Using the adjustments and tuning described above, we can measure the isotope ratios of secondary ions, which is at the core of MIMS methodology, in a precise and reproducible manner.

### Spatial resolution

The parallel detection of different ions, the small size of the primary-ion beam, and the high efficiency of secondary-ion collection all contribute to the highly effective spatial resolution of MIMS, reaching 33 nm, 30-fold better than in previous instruments. Smaller beams are possible and may be useful for locating and measuring highly concentrated tags in volumes of the order of (20 nm)^3^. Primary sources with higher brightness could give smaller probes with sufficient current for practical analysis: they could be provided by a brighter Cs^+ ^primary-ion source or by other types of sources [4].

### Volume sputtered and detectability limit

The absolute sensitivity of SIMS is among the highest of current chemical analysis methods and the total amount of material needed for MIMS analysis is extremely small. Using MIMS, one can detect parts per billion of an isotope in a sub-cubic-micrometer volume of sample. Let us assume an analyzed field of 6 × 6 μm, a primary Cs^+ ^beam current intensity of about 0.4 pA, a dwell time per pixel of 20 msecs, 256 × 256 pixels, a sputtering efficiency of five target atoms per Cs^+ ^ion, an atomic layer thickness of 2.55 × 10^-4 ^μm, and an atomic density of 6 × 10^10 ^atoms per μm^3^: the total sputtered volume is then 0.27 μm^3^, and the sputtering rate is 0.4 nm/minute or 1.5 atomic layers/minute. For a probability of detection of 95%, three ions have to be emitted; assuming a useful ionization yield of 1 × 10^-2 ^ions/atom, then the minimum detectable amount is 1.85 × 10^-8 ^or approximately 20 parts per billion (see Additional data file 1).

### Contrast formation

There is no theory using first principles that allows us to predict what happens when the surface of the sample is hit by a primary ion. Which atoms are most likely to escape?

What is the sputtering rate (the rate of removal of target atoms)? When a given atomic species is sputtered, what is the ionization yield (the fraction of atoms that are ionized)? How will the composition of the matrix affect the number of atoms sputtered, the ionization yield, and the formation of polyatomic clusters such as CN or CNH? What is the breaking pattern of large and small molecules in atoms and atomic clusters? How interdependent are the parameters mentioned? All these questions are yet to be answered. In addition, the choice of primary ions, so important in practice, remains mostly empirical. Indeed, in order to achieve the high ion yields required for trace analysis down to the low parts per billion range, the sample needs to be bombarded with or exposed to chemically reactive species, and O^- ^or Cs^+ ^are most commonly used. There is, however, a lack of detailed knowledge about the instantaneous surface composition, and all attempts to correlate observed ion yields with available ionization models should be considered highly questionable [39].

The contrast of a mass image is due to regional differences in secondary-ion currents emitted over the analyzed field. The main determinants of secondary-ion formation are the sputtering rate (the rate of removal of the target atom by the primary ions), the ionization yield (the fraction of sputtered atoms that are ionized) and the local concentration of atoms. These parameters cannot be derived directly from first principles, but we may safely assert the following. The sputtering rate in steady state is directly proportional to the primary beam current density at the sample surface and is dependent on the local atomic composition.

The ionization yield, in the negative secondary-ion emission mode, depends strongly on both the electron affinity of the elements or clusters under consideration and the concentration of the Cs^+ ^ions that are implanted by the beam into the superficial atomic layers of the sample. To a first approximation, implanted primary-ion concentrations vary as the inverse of sputtering rates; this makes the ionization yield somewhat a function of the sputtering rate. However, we do not know the form of the function that links implanted Cs^+ ^concentration to ionization yield.

Taking all these factors in consideration, we can predict the following. A low sputtering rate is generally associated with a higher concentration of implanted Cs^+^; lower sputtering rates should therefore lead to higher ionization yields. If ionization yields are strongly dependent on Cs^+ ^concentrations, low sputtering rates may paradoxically lead to higher secondary-ion currents. In a situation in which the sputtering rates of different areas over the field of analysis are not the same, a given area may appear brighter because the sputtering rate is lower (with a relatively higher concentration of implanted Cs^+^) and the ionization yield is still sensitive to the concentration of implanted Cs^+^. Moreover, the ionization yield is likely to depend on local sputtering rates because, once a steady state has been reached, these local rates control the local concentrations of the implanted primary ions. As a consequence, the brightness of an image area depends upon the local concentration of the element under consideration, the implanted Cs^+ ^concentration and the sputtering rate.

Without any contrast, images would not exist. Parallel mass images of different elements with different electron affinities convey a lot of information. For instance, a section of embedded cells bombarded with Cs^+ ^gives ^12^C^- ^images with low contrasts and, in parallel, ^12^C^14^N^- ^images showing strong contrasts related to known cell structures (for instance, Figure 14, ^12^C^- ^and ^12^C^14^N^-^). Let us first consider ^12^C^- ^images. The local ^12^C^- ^intensity depends on both the sputtering rate and the ionization yield. When analyzed in parallel, one would expect (but it is not demonstrated) ionization yields of ^12^C^14^N^- ^ions, with their high electron affinity (3.6 eV), to saturate (to reach their maximum electron capture probability) more rapidly during Cs^+ ^implantation than ^12^C^- ^ions, whose electron affinity is much lower (1.2 eV). It follows that, if the ionization yield is strongly dependent on the concentration of implanted Cs^+^, at low concentrations of implanted Cs^+ ^the ^12^C^- ^ion emission should be more sensitive to small changes in the implanted Cs^+ ^and may be more sensitive than ^12^C^14^N^- ^ions to local differences or fluctuations in sputtering rate.

The low contrast that we observe in ^12^C^- ^images of epon-embedded samples probably indicates that sputtering rates are evenly distributed across the field analyzed and that implanted Cs^+ ^concentrations are approximately equivalent throughout the sample. The remaining contrasts are probably due to a slightly lower concentration of carbon in the biological tissue than in the embedding epon and to the formation of ^12^C^14^N atomic clusters that reduce the total number of ^12^C atoms available to form ^12^C^- ^ions. If high contrasts were observed it would alert us to differences in sputtering rates or unexpected differences in atomic concentrations of carbon.

Because ^12^C^- ^images indicate a homogeneity in the sputtering rate and a steady state of the implanted Cs^+ ^concentration, the contrast observed on the CN^- ^images acquired in parallel must reflect differences in local nitrogen concentration over the analyzed field and cannot be due to emission artifacts. The fact that, in many instances, interpretation of ^12^C^14^N^- ^images is straightforward and allows us to recognize easily details of features in tissue or cell sections is a boon.

Finally, we wish to emphasize the strength of the quantitative information derived from the ratio of mass images acquired in parallel. All parameters being the same, atoms or clusters such as ^26^CN and ^27^CN are affected identically at a level of precision well beyond what is needed for our experiments, and the quantitative ratio images contain the true isotope ratio values, cancelling out sputtering, ionization and other effects that affect the emission of a single ion.

### The relationship between contrast formation, lateral resolution and counting statistics

The lateral resolution is essentially a function of the size of the primary ion beam. The smaller the beam diameter, the higher the resolution; to be visually appreciated, however, the image has to have enough contrast; that is, the image has to contain enough counts. Higher counting rates will be obtained with a brighter primary ion beam, all other conditions being the same. The count rate does not affect the intrinsic resolution, but it is a main determinant of the counting statistics. For example, in a parallel set of ^12^C^15^N^- ^and ^12^C^14^N^- ^images, although a ^12^C^15^N^- ^region in general contains far fewer counts than its ^12^C^14^N^- ^homolog, it has the same lateral resolution. The variance of the ^12^C^15^N^-^/^12^C^14^N^- ^isotope ratio, however, is essentially determined by the number of counts in the ^12 ^C^15^N^- ^region.

If the sample surface being studied is not flat but shows relief, we observe a three-dimensional appearance in the sample image. Here, the contrast may be due either to a differential implantation or to a differential sputtering that occurs because the primary beam attacks the sample surface at a variety of angles. It should be emphasized, however, that these phenomenon affect in the same proportion – at least to a first approximation – isotopes separated by 1 atomic mass unit. Thus, the variations in count rates cancel out (note that all relief effects disappear in the ratio images; see for example the adipocyte in Figure 4d,f), and the value of the isotope ratio still represents the local abundance of the individual isotopes with a precision of a few percent, which is entirely satisfactory for biological purposes. SIMS had previously been used for quantitation in the absence of imaging, and for imaging in the absence of quantitation. The advantage of MIMS is to combine high spatial resolution (that is, analyzing a much smaller amount of material than previously), with simultaneous (parallel) acquisition of information from several atomic masses, and with isobar separation at mass 27.

The ability to provide quantitative information is a unique attribute of MIMS. Imaging is a guide for exploiting the massive amount of unique quantitative information, which is based on the mining of the counts of secondary ions per pixel.

### Isotope ratios

The isotope ratio values are the essential data needed to interpret the results, and the derivation of isotope ratios is an integral part of MIMS methodology. The absolute number of counts for a given atomic mass do not convey any information about the presence of an isotope excess. For example, the counts of ^12^C^15^N by themselves do not allow us to conclude anything about the presence of a ^15^N tag in the analyzed region. Only the ratio ^15^N/^14^N compared with the natural abundance of ^15^N is informative. The visual observation of contrast and/or brightness is no more informative, and may be misleading. In a given field, contrast is due to variations of the number of counts per pixel, which may have multiple undocumented causes. As discussed under 'Contrast formation' earlier, in addition to the local abundance of the isotope under consideration, the secondary-ion intensity depends on other parameters such as the number of atoms of Cs implanted and the quality of the matrix into which the isotope is introduced. Thus, the fact that a given area emits more than a neighboring one does not mean that it contains more of the isotope tag. High counts in ^12^C^15^N do not mean that there is an excess of ^15^N until they are normalized with respect of the counts of ^14^N by calculating the isotope ratio^12^C^15^N/^12^C^14^N. A region of high secondary-ion yield at mass ^12^C^14^N may have little incorporation of ^15^N (see, for example, the cochlear outer pillar cells (Figure 3a), which have a high secondary ^12^C^14^N yield and very low incorporation of ^15^N (Figure 3c and Additional data file 3). Conversely, a region with high ^15^N on the ^12^C^15^N mass image compared with the neighboring pixels may simply reflect a region with overall high yield of both ^12^C^14^N and ^12^C^15^N, even though their proportion is equivalent to the natural ratio. The isotope ratio values for regions of interest are the only information that may be used for evaluating where and how much a label such as ^15^N has been incorporated.

The power of the isotope ratio method is that all analytical parameters being the same (field analyzed, instrumental conditions, Cs^+ ^implantation, matrix effect), one may assume that they affect two isotopes of the same atomic number – that is, isotopes that differ in general by 2 atomic mass units at most – in an identical fractional manner, so that any unknown effect will cancel out and that isotope ratio values will uniquely represent the relative abundance of one isotope with respect to the other.

Ensuring 'flatness' of the field, such that the isotope ratio values among regions of interest are equivalent regardless of their location in a control image field, is very important. It demonstrates that there is no differential fractionation among isobars: that is, that differences in isotope ratio among various areas are not the consequence of changes in transmission between a set of isotopes that are related to location within the field. This is why the ^12^C and ^13^C quantitative atomic mass images used to generate the ^13^C/^12^C ratio image (in Figure 3, for example) are an essential internal control. Because there is no ^13^C added, they control for the flatness of the field, which means: first, equivalence of the ^13^C/^12^C ratio at any location over the field analyzed; second, an absence of instrumental drift during analysis; third, the lack of some topographical effect; and fourth, the lack of a differential matrix effect. With all conditions being the same and because the secondary ions are detected in parallel from the same analyzed area, this ensures that measured high values of ^15^N/^14^N ratios do show an excess of ^15^N and cannot be an artifact.

We have developed several kinds of controls. Two essential controls in our quantitative imaging allow us to validate the analytical conditions. This validation is individual to the field being analyzed. The first control is to make sure that if no isotope has been added, the isotope ratio measured is equivalent to the natural abundance in a region of the field where no excess isotope is expected. When the sample is an epon-embedded section, this is measured on the epon, in a region of the analyzed field that does not contain tissue or cells.

The second essential control is to make sure that, for every field analyzed in quantitative imaging mode, a calculated excess of an isotope above its natural abundance is independent from its location in the analyzed field. This could happen if there was some instrumental fractionation of one isotope with respect to another, meaning that depending upon the location over the field, the transmission for one of the isotopes is somewhat different from another one. Control for the lack of fractionation is provided by the image derived from isotope ratios from quantitative mass images of a set of isotopes expected to be present at their natural abundance values and recorded over the same field, in the same instrumental condition and in parallel, simultaneously, with the isotope from which one expects an excess above its natural abundance at some location within the field. This is best performed by recording together in parallel the quantitative image of the experimental isotope and of an isotope that is expected to be present at the natural abundance level. The beauty of the ^13^C/^12^C ratio (as in the cochlear sample labeled with ^15^N, Figure 3) or of the ^12^C^15^N/^12^C^14^N compared with ^13^C^14^N/^12^C^14^N (as in an adipocyte labeled with ^13^C, Figure 4) is that they control for the very same field that is being analyzed, and in the same conditions. 'Normally' one is not fortunate enough to develop and use internal controls of such high power. These controls are ideal internal controls and are immensely more important than finding natural abundance value ratios in separate control samples where all conditions are different from the experimental measures.

## Conclusion

We have developed MIMS, combining the use of a new SIMS instrument that simultaneously detects multiple isotopes with stable isotope labeling and quantitative image analysis. Using MIMS, it is possible to localize and measure precisely stable or radioactive isotopic tags in subcellular compartments in sub-cubic-micrometer volumes. Stable isotopes can be localized simultaneously at high resolution and measured with high precision, a unique combination. Studies with MIMS can use conventional tissue – and cell-preparation methods. The molecular tags can be any isotopes, stable or radioactive. One can take advantage of any molecule that has been labeled for mass spectrometry or nuclear magnetic resonance studies (^2^H, ^13^C, ^15^N, ^17^O, ^18^O, ^33^S) or for radioactive studies (^3^H and ^14^C). These features make MIMS methodology a powerful new way of viewing and measuring, in all fields of biomedical research, important parameters that were impossible or difficult to see or to measure with other methods.

## Materials and methods

Data analysis was performed with an SGI O2 workstation, ISee Analytical Imaging Software (Inovision Corporation, Raleigh, NC), custom software within ISee Analytical Imaging Software, and Prophet statistical software (National Center for Research Resources, NIH).

### Quantitative image data extraction

In the scanning mode, the number of counts for each secondary ion emitted from each pixel is stored in a computer file. Grayscale secondary-ion images for each of the selected secondary ions are obtained after analysis by reconstructing the data onto an array with a number of pixels equal to that of the data-acquisition array, with the pixel intensity in the image scaled according to the number of counts detected from that position for the ion. We developed a piece of software to extract 24-bit quantitative image files from hundreds of raw MIMS images, keeping the counts recorded in each pixel.

An essential feature of MIMS is the ability to locate and to quantitate regions of the sample that have accumulated an isotope, for example ^15^N, after the isotope has been provided externally. This is done by deriving ratio images. For example, the ratio image ^12^C^15^N^-^/^12^C^14^N^- ^and the ratio image ^13^C^-^/^12^C^- ^are derived from the pixel-by-pixel division of the respective parallel images. Quantitative images acquired with MIMS generate far more information than can be displayed using grayscale methods. To show high dynamic-range ratio images and to de-emphasize values resulting from data with few counts, we developed a method based on a hue saturation intensity transformation (HSI) [40,41] of the ratio image; HSI is derived from a calcium ratio imaging procedure [42]. Think of the pixels in a MIMS image as equivalent to test tubes. A 256 × 256 pixel MIMS image would be an array of 65,536 test tubes from which we simultaneously count several isotopes (for example, the abundance of ^12^C, ^13^C, ^14^N and ^15^N). The HSI method allows us to identify among the 65,536 pixel test tubes (corresponding to areas of sub-micrometer size) those that contain significantly higher label counts. The main advantage of HSI is to color code the value of the ratios following a scale that can be extended or compressed, as well as taking full advantage of the fact that humans perceive many more differences in color than differences in shades of gray. For example, HSI enables us to identify regions of interest with significant excess of the ^15^N label by a means independent of visual recognition of expected histological structures. The use of HSI allows us to greatly simplify the first step of data analysis.

Another powerful piece of software automates the data analysis of sequences of raw images. After creating stacks of images from analyzed fields and drawing regions of interest, statistics for each of the regions in each of the images are automatically tabulated and exported to the Prophet software for statistical analysis.

### Isotope ratios

Regions of interest were selected using HSI ratio images; secondary-ion counts for the same regions of interest were extracted from the corresponding unsmoothed images of ^12^C^-^, ^13^C^-^, ^12^C^15^N^- ^and ^12^C^14^N^- ^and the isotope ratio values from the derived images ^13^C^-^/^12^C^- ^and ^12^C^15^N^-^/^12^C^14^N^-^. The ^15^N/^14^N ratios (or ^13^C^-^/^12^C^-^) for each region of interest were also determined by dividing the total counts of ^12^C^15^N^- ^(or ^13^C^-^) over all pixels in that region by the total corresponding counts of ^12^C^14^N^- ^(or ^12^C^-^). Importantly, none of the image manipulations that we perform alters the counts contained in the pixel or groups of pixels.

### Nitrogen renewal

The percent nitrogen renewal was calculated for each region of interest as: 100 × (tissue ^15^N/^14^N ratio – control diet ratio)/(experimental diet ratio – control diet ratio), which gives the percentage of the labeled dietary nitrogen that has been incorporated into the sample. Zero percent represents a ratio equivalent to the terrestrial ratio; 100% represents a ratio identical to that in the experimental diet.

### Labeling and tissue preparation

All experimental protocols used in this study were approved by the Harvard Medical Area Standing Committee on Animals, Protocol 02751. CBA/CaJ mice, 8 weeks old at the start of the experiments, were obtained from Jackson Laboratories (Bar Harbor, ME, USA).

### Diets enriched in ^15^N-leucine

Standard L-leucine commercial protein-free amino acid diet (TD 86529, Harlan Teklad, Madison, WI, USA) was used as the control diet. The experimental diet was prepared by the manufacturer according to the same formula, but with ^15^N-L-leucine replacing 23% of the standard L-leucine (9 g ^15^N-L-leucine in the total of 38.8 g leucine, for a 3.5 kg batch of diet). To ensure a dietary steady state on these synthetic diets, mice were placed on the control amino-acid diet for 5 days before they were fed the experimental diet. Mice were sacrificed after 9 days or 22 days on the ^15^N-L-leucine diet. ^15^N-L-leucine (> 98% isotopically pure) was obtained from Cambridge Isotope Laboratories, Andover, MA, USA.

The ^15^N/^14^N nitrogen isotope ratios in the diets were measured by combustion mass spectrometry analysis (Metabolic Solutions, Nashua, NH, USA). The values were 3.657‰ (0.001 SE, *n *= 12) in the control diet (equivalent to the terrestrial value) and 13.972‰ (0.573 SE, *n *= 12) in the experimental diet.

### Mouse cochlear tissue

After 9 or 22 days on the diet, deeply anesthetized mice underwent intracardiac perfusion with saline followed by fixative (2.5% glutaraldehyde and 1.5% paraformaldehyde in 0.065 M phosphate buffer). The temporal bones were removed, kept in fixative overnight at 4°C, decalcified (0.1 M EDTA with 0.4% glutaraldehyde) for 3 days, rinsed in deionized water, dehydrated in ethanol and propylene oxide, infused with propylene oxide/epon, and embedded in epon. Other tissues were treated similarly, but not decalcified [43].

Sections from the epon-embedded tissue, 0.5 mm in thickness, were cut with glass knives on a Sorvall ultramicrotome. The sections were deposited on (5 mm)^2 ^silicon wafer chips, which provide a mechanically sturdy and adequately conductive support [44]. The silicon chips were then mounted in a custom-made sample holder for insertion into the MIMS instrument. The holder with the sections was placed in a vacuum oven for 3 days at 50°C before analysis and then introduced in the preanalysis chamber of the instrument in a 10^-9 ^torr vacuum.

#### Teredinibacter turnerae

Cells of *T. turnerae *were grown in sealed 16 ml Hungate tubes containing 15.5 ml of simple salt medium (SBM amended with 0.5% sucrose without combined nitrogen [31]) allowing a headspace of 0.5 ml of atmospheric gas. After three days the headspace gas was removed by displacement with fresh medium and replaced with 0.5 ml of ^15^N gas (99%). Cells were then incubated at 22°C with gentle shaking for 0, 0.25, 0.5, 1, 2, 4, 8, 16, and 32 h. After incubation, cells were sedimented by centrifugation (12,000 *g*, 10 min, 4°C) and fixed by resuspension in 500 ml 3.7% formaldehyde in seawater for 30 min. Cells were then washed by sedimentation (14,000 *g*, 10 min, room temperature) and resuspended first in 50% ethanol and again in distilled water. Cells were finally resuspended in 10 μl of distilled water and 2 μl was applied to the silica wafer and air-dried at 37°C. Cells of *Enterococcus faecalis *were prepared identically except that they were grown in Tryptic Soy Broth medium (Bacto) at 37°C for 2.5 h (approximately eight doublings).

### Shipworms

Shipworms, *Lyrodus pedicellatus*, were incubated in seawater in a ^15^N atmosphere, fixed in formaldehyde, embedded in epon, and sectioned for MIMS analysis.

### Adipocytes

Adipocytes were prepared and analyzed in MIMS using the same methods as Kleinfeld *et al*. [28].

### Rat embryo fibroblasts

Rat embryo fibroblasts (REF52) were cultured in DME/F12 medium with 10% FCS. They were serum-deprived for 26 h after they reached 70–80% confluence; pulsed for 17 h with 10% FCS and 1 mM BrdU (1/10 of the concentration generally used in microfluorescence studies) and 1 mM ^15^N-uridine; fixed in their culture dish (using formaldehyde, glutaraldehyde, and picric acid); detached from plastic with propylene oxide; embedded in epon-Araldite; sectioned at 0.5 μm thickness; and mounted on polished Inox rod for MIMS analysis.

For ^14^C analysis, REF52 cells were cultured in DME/F12 media with 10% FCS. They were serum-deprived for 24 h after they reached 40% confluence; pulsed for 24 h with 10% FCS and 1 mCi ^14^C-thymidine at a concentration of 19.2 nanomol/ml; fixed in their culture dish using formaldehyde and glutaraldehyde; detached from plastic with propylene oxide; embedded in epon-Araldite; sectioned at 0.5 μm thickness; and mounted on silicon chips for analysis.

### Tuning for quantitative mass image acquisition

Before a MIMS experiment, the sample is first viewed and photographed under a reflection differential interference contrast (RDIC) microscope with an automated stage, and the coordinates of multiple regions of interest are noted and used to find the same regions in the automated stage of the SIMS instrument. RDIC allows us to dispense with preparing and staining alternate sections for microscopy. The sample is placed in the analysis chamber under a vacuum of approximately 3 × 10^-10 ^torr. A primary-ion beam of Cs^+^, after being shaped by the primary-ion column, is focused with the immersion lens and strikes the sample with an energy of 16 keV (see Figure 13). The primary-ion beam scans the region of interest on the stationary sample step-by-step in a raster pattern, generally of 256 × 256 pixels but the number of pixels can vary in practice from 32 × 32 to 1,024 × 1,024. The secondary ions emitted from the sample are focused with the immersion lens, shaped by the optics of the secondary-ion column, and sent through a hexapole in the double-sector mass spectrometer made up of an electrostatic prism followed by a magnetic prism. This permits first-order focusing of the secondary ions as narrow lines along the focal plane of the magnet. Four detector units are located at the focal plane and can be moved independently along it so as to select specific atomic masses. Four electron multipliers in the detector units detect the signals from the selected secondary ions simultaneously. The current and diameter of the primary-ion beam, the time the beam is directed at each pixel (the dwell time), and the size of the pixels can be adjusted according to the size of the area being analyzed.

For each analysis, we need to ensure first that we have implanted enough Cs atoms into the sample to maximize the secondary-ion counting statistics; second, we maximize the secondary-ion transmission; third, we have perfect mass spectra at all masses; and fourth, in control fields we obtain equivalent values of the isotope ratio over the entire image field. Tuning is first done in ion-counting mode, in which the secondary-ion count rate recorded by each detector is displayed numerically. No images are acquired. We ensure that the responses of the electron multiplier detectors (EM; see Figure 13) are equivalent. The primary Cs^+ ^beam is defocused and kept stationary on a standard silicon sample. The secondary-ion current at mass ^28^Si is measured successively with each of the four EM detectors; the radius of the secondary-ion beam is set to enter the appropriate EM by changing the magnetic field strength of the magnetic prism. We adjust two parameters for each EM: the EM high voltage is set so that the pulse height distribution (PHD, the graphical representation of the distribution of the amplitude of the pulses generated by an EM at a particular voltage) of the EM output is similar for all four detectors; and the EM threshold voltage is set so that, in the absence of secondary-ion current, the noise of the counting system is smaller than 1 count per minute. When these conditions are fulfilled, the count rate for the same sample is the same among the four detectors, within ± 2.5%.

To begin tuning, we implant Cs atoms into the biological sample over a relatively large field (100–150 μm) at high primary-ion current (obtained by removing the diaphragm (D1, Figure 13) used to define the primary-beam aperture in the objective column (OC). After a period of several minutes, Cs atoms implanted into the sample enhance the secondary-ion yield so that the ^12^C^14^N^- ^secondary-ion count rate reaches a steady state. Next, we ensure that the EM detectors are located in the correct position along the focal plane of the magnet (FP, Figure 13) for each mass. We maximize the count rate of each EM by moving the detector along the focal plane so as to maximize the secondary-ion count rate for the selected atomic mass.

We follow with the alignment of the secondary-ion beam so that the isobars ^12^C^15^N and ^13^C^14^N, and ^13^C and ^12^C^1^H, are well discriminated, indicating high mass resolution, while maintaining a high secondary-ion transmission (approximately 70–80%). An entrance slit (ES, Figure 13), whose width is a main determinant of the mass resolving power (MRP), is placed in the path of the secondary-ion beam. We maximize the count rate for a given mass by iteratively focusing the secondary-ion lens located in the objective column (E0S) and by adjusting the voltages of the deflection plates P2, P3, and CY within the secondary column. We then set an aperture slit (AS) and an energy slit (WS) to reduce angular and energy aberrations and thus to maximize the MRP. To evaluate the MRP, we scan the secondary-ion beam through the detectors selection slit (SS) using a set of deflection plates (DP) and we record a small portion of the mass spectrum by plotting the secondary-ion current versus the deflection voltage (Figure 15). The quadrupole and slit lens (LF4) are used to maximize the MRP. Then, for each detector, using the mass spectra plots, we set the voltage of the deflection plates (DP, Figure 13) so that the mass line of interest is centered in the selection slit (SS) of the EM (see Figure 13). This practically eliminates any spillover from neighboring isobars.

We then switch to imaging mode, which displays 'live' scanning ion images from two selected masses. After adjusting the size of the analyzed field, the last steps aim to balance the tuning and to focus the primary-ion beam. We ensure that the analyzed field is balanced, that is, that the secondary-ion intensity emitted by a small fraction of the field is equivalent over the entire field, with iterative small voltage adjustments of the secondary-ion objective lens (EOS), and of a series of deflection plates (P2, P3 and CY) within the secondary column. We optimize the focus by adjusting the voltage of the primary-ion objective lens (EOP, Figure 13). To analyze at the highest possible spatial resolution (approximately 35 nm), we increase the demagnification of the primary-ion source by increasing the voltage of the lens L1 (see Figure 13) in the primary column. We may then have to slightly readjust the tuning of the secondary-ion beam. Finally, before starting analysis, we adjust the number of pixels in the image (256 × 256 up to 2,048 × 2,048) such that the beam diameter is approximately twice the pixel size, and we ensure that the acquisition time is long enough to obtain sufficient counting statistics for masses with low count rates (for example ^13^C and ^12^C^15^N).

After a sample is introduced into the MIMS analysis chamber, we can set the instrument to acquire qualitative MIMS images – in general at mass ^12^C^14^N, which gives a good anatomical view – within 30 minutes. Significantly more time is required, however, for the optimization of the many instrumental parameters (tuning) that is needed to record quantitative mass images suitable for extracting meaningful quantitative data. For new types of samples it may take hours to a day. Yet the physics is robust, and further tuning on an identical type of sample does not take more than 1 to 2 hours.

## Additional data files

The following files are available: Additional data file 1, containing an explanation of how the volume sputtered, the useful yield, and the detectability limit are calculated; Additional data file 2, containing an explanation of how the lateral resolution is calculated; Additional file 3, describing data reduction from labeled and control mouse cochlear sections; Additional data file 4, describing the relationship between the brightness of an image and the number of counts; Additional data file 5, comparing MIMS with autoradiography; Additional data file 6, containing an explanation of the shape of mass line peaks in MIMS; and Additional data file 7, containing more details on Figure 2e.

## Supplementary Material

Additional data file 1 An explanation of how the volume sputtered, the useful yield, and the detectability limit are calculated Click here for additional data file

Additional data file 2 An explanation of how the lateral resolution is calculated Click here for additional data file

Additional data file 3 Data reduction from labeled and control mouse cochlear sections Click here for additional data file

Additional data file 4 The relationship between the brightness of an image and the number of counts Click here for additional data file

Additional data file 5 A comparison of MIMS with autoradiography Click here for additional data file

Additional data file 6 An explanation of the shape of mass line peaks in MIMS Click here for additional data file

Additional data file 7 More details on Figure 2e Click here for additional data file
