# Supplementary material for: High-resolution quantitative imaging of mammalian and bacterial cells using stable isotope mass spectrometry
Source: J Biol. 2006 Oct 5;5(6):20. doi: 10.1186/jbiol42 (PMC1781526; doi:10.1186/jbiol42)
Supplement: Additional data file 1 — An explanation of how the volume sputtered, the useful yield, and the detectability limit are calculated [file jbiol42-s1.pdf]

Additional File 1: Estimation of the volume sputtered, useful ion yield and detectability limit

We will illustrate below how we will estimate the volume sputtered, the useful yield and the detectability limit.

Field analyzed	6 μm x 6 μm = 36 μm^2
Cs^+ beam intensity	~ 0.40 pA
or	2.5 x 10^6 ions $Cs^+ s^{-1}$
Dwell time/pixel	20 ms
Number of pixels	256 x 256
Acquisition time	1310 s
Total dose (TD)	3.27 x 10^9 Cs^+ ions
Dose/μm^2	9.10 x 10^7 Cs^+ ions
Dose / surface atom	5.94 **~ 6 Cs^+ ions / surface atom**
Sputtering efficiency[a]	5 target atoms / Cs^+
→ Total number of atoms sputtered[b]	1.64 x 10^{10}
Atomic density[c]	6.00 x 10^{10} atoms / μm^3
Thickness of one atomic layer	2.55 x10^{-4} μm
Number of atoms / μm^2 in one layer	1.53 x 10^7
→ Sputtered volume for TD	0.27 μm^3 (1)
or Sputtered thickness	7.59 x 10^{-3} μm **~ 8 nm**
or Atomic layers	2.97 x 10^1 **~ 30 layers**

a Supported by many measurements in material sciences. The measured values approximately range between 3 target atoms/Cs^+ for ^{12}C to 20 target atoms/Cs^+ for ^{197}Au.

b For one series of parallel images.

c Lower estimate calculated from concentration of H, N, O and P in dry biological tissue and excluding embedding medium.

The useful ion yield for detecting nitrogen can be estimated; in the above conditions

Dose / pixel	~ 5.00×10^{4} Cs^{+} ions;
they sputter	2.5×10^{5} atoms / pixel
Nitrogen atomic concentration in the sample[a]	5.00×10^{-2}
→ Nitrogen atoms sputtered / pixel:	1.25×10^{4}
Signal intensity for $^{12}C^{14}N^{-}$	2000 ions / pixel[b]
→ Useful yield	**1.6×10^{-1} CN^{-} ion / target N atom**

Minimum detectable:

Atomic density	6.00×10^{10} atoms / μm^{3}	
Useful yield[c]	1.0×10^{-1} ion / atom	
30 sputtered atoms	→ 3.0 ions (mean)	
probability of 0 ion detected	5%	
probability for detecting a least 1 ion	95%	
From (1), volume analyzed	0.27 μm^{3}	
Number of atoms	1.62×10^{10}	
Minimum detectable	1.85×10^{-9}	**~ 2 ppb**

---

a Estimated from concentrations of H,C, N, O, P in dry biological tissues.
b Experimental data. Preliminary results.
c Under estimate from our experimental conditions.

Assume now,

Analyzed diameter	100 nm	
Thickness	10 nm	
→ Volume	7.85×10^{-5} μm^3	
Number of atoms	4.71×10^{6}	
Minimum detectable	6.37×10^{-6}	**~ 7 ppm**
