# Supplementary material for: High-resolution quantitative imaging of mammalian and bacterial cells using stable isotope mass spectrometry
Source: J Biol. 2006 Oct 5;5(6):20. doi: 10.1186/jbiol42 (PMC1781526; doi:10.1186/jbiol42)
Supplement: Additional data file 2 — An explanation of how the lateral resolution is calculated [file jbiol42-s2.pdf]

Additional File 2: Estimation of lateral resolution

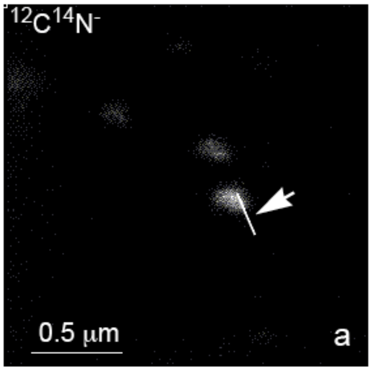

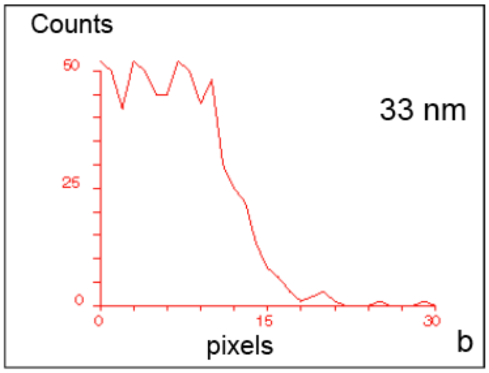

We have estimated a spatial resolution from the $^{12}C^{14}N^-$ signal of a 'dust grain' scanned over a 2 µm x 2 µm area, 256 x 256 pixels. The intensity count profile along the line indicated by the arrow in panel a is shown in panel b. Using the '16%-84% maximum signal' criterion, the calculated lateral resolution is 33 nm. The real resolution is likely to be better because the sample – and therefore the step function – are not ideal. Note that the critical aspect of the measurement is ensuring that on either side of the sharp edge, the "knife edge", one has a relatively constant intensity (flat bottom and flat top) over at least twice the estimated diameter of the probe size. See: Michael, J.R. and D.B. Williams, A consistent definition of probe size and spatial resolution in the analytical electron microscope. Journal of Microscopy, 1987. **147** Pt. 3: p. 289-303.
