# Supplementary material for: High-resolution quantitative imaging of mammalian and bacterial cells using stable isotope mass spectrometry
Source: J Biol. 2006 Oct 5;5(6):20. doi: 10.1186/jbiol42 (PMC1781526; doi:10.1186/jbiol42)
Supplement: Additional data file 3 — Data reduction from labeled and control mouse cochlear sections [file jbiol42-s3.pdf]

Additional File 3

Data reduction

We present the data reduction for the analysis of a single field - corresponding to the images shown in Figure 3a-f - that leads to the values of ^{15}N incorporation in a variety of cochlear structures. We also present data from a control sample from this set of experiments. We use a quantitative image analysis software (Isee, Inovision Corporation, Raleigh, USA) with many additional custom pieces of software.

From this single field image acquisition, we first extract seven image files: the four original quantitative mass images (^{12}C, ^{13}C, $^{12}C^{14}N$, and $^{12}C^{15}N$), two derived ratio images ($^{13}C/^{12}C$ and $^{12}C^{15}N/^{12}C^{14}N$; Figure 3a-f), and the hue saturation intensity (HSI) transform of the $^{12}C^{15}N/^{12}C^{14}N$ isotope ratio (Additional Figure 1a**)**. One can study a field with the HSI image software to uncover regions of interest, using many functions - thresholds, transparency, medianization etc. - contracting or expanding ratios between a variety of low and high limits and displayed following the rainbow colors.

We stack the four mass images, the two derived ratios images and the HSI image. We select regions of interest (ROI) by drawing manually on the HSI image and/or on the mass $^{12}C^{14}N$ image (Additional Figure 1a-b; in the present example, 53 regions). Then, with another piece of custom software, we index the ROI and generate a matrix containing the statistics of the ion counts at each ROI for all the quantitative mass images and the derived ratio images. A portion of this matrix, showing the ROI index, the number of pixels, the mean ^{26}CN and ^{27}CN counts, their standard deviations, the sum of the counts, and the $^{27}CN/^{26}CN$ ratio values, in all 53 ROIs, is presented in Additional Table 1. Not shown is the similar data for ^{12}C, ^{13}C, and the $^{13}C/^{12}C$ ratios.

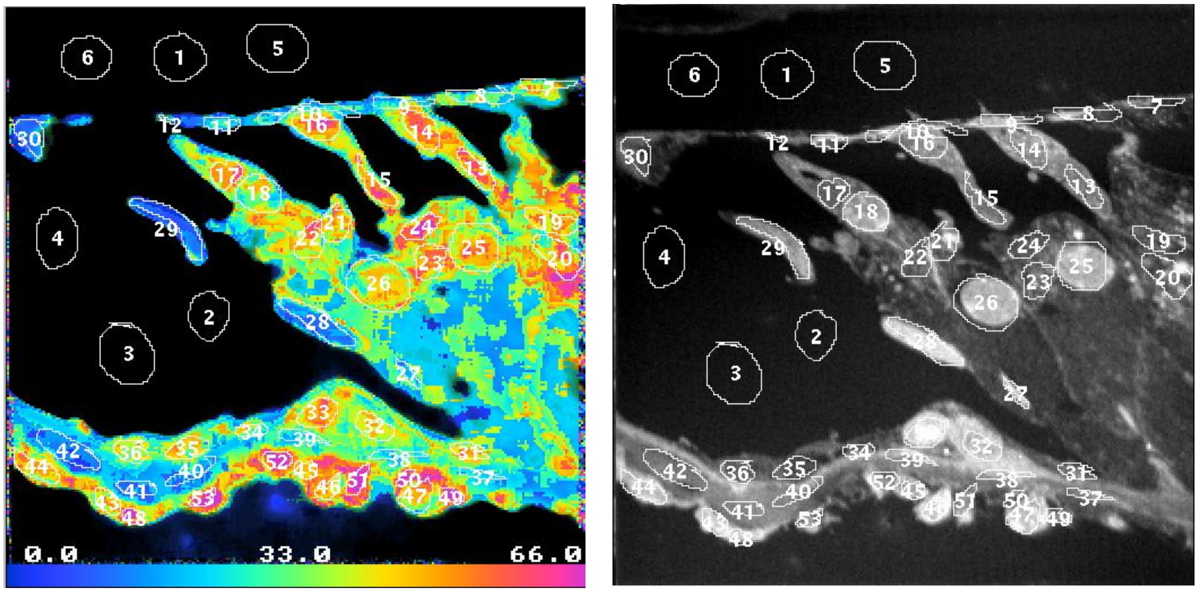

(a) **(b)**

Additional Figure 1
(a) HSI transform image of the $^{12}C^{15}N/^{12}C^{14}N$ ratio from the mouse cochlear section shown in Figure 3c. The field of view is 60 µm x 60 µm. The scale represents the percentage ^{15}N renewal; see the formula in the legend to Additional Table 2. **(b)** $^{12}C^{14}N^-$ image of the same area. We have drawn 53 ROIs around cellular structures of the cochlea. Note that there is no correlation between the contrast in the $^{12}C^{14}N$ image and ^{15}N incorporation, as seen in the HSI image (a). For example, ROI number 28, corresponding to an outer pillar cell, appears bright in the $^{12}C^{14}N$ image (additional figure 1-b) and in

the $^{12}C^{15}N$ image (figure 3b) but its $^{12}C^{15}N/^{12}C^{14}N$ value is relatively low, 55.3 (additional table 1, row ROI #28), giving a low value for ^{15}N renewal 18.1%, visible as blue green in the HSI $^{12}C^{15}N/^{12}C^{14}N$ ratio image (additional figure 1-a).

Additional Table 1
A portion of the statistical data derived from the ROIs shown in additional figure 1

ROI	n Pix	^{26}CN Mean	^{26}CN sd	^{26}CN Sum	^{27}CN Mean	^{27}CN sd	^{27}CN Sum	^{27}CN/^{26}CN "Mean"	^{27}CN /^{26}CN "Sum "
1	411	351.8	21.2	144608	1.2	1.1	477	33.1	33.0
2	326	490.2	26.0	159796	1.8	1.6	601	37.7	37.6
3	563	471.8	27.2	265619	1.9	1.7	1091	41.1	41.1
4	428	324.1	30.1	138708	1.2	1.3	523	38.2	37.7
5	513	421.4	23.5	216158	1.4	1.4	743	34.4	34.4
6	374	287.0	21.2	107328	1.0	1.2	365	34.3	34.0
7	109	848.4	231.0	92476	7.5	4.2	812	85.2	87.9
8	174	943.6	325.4	164191	6.7	4.2	1174	69.3	71.5
9	119	1285	343	152940	9.1	4.6	1086	68.6	71.0
10	173	1067	348	184606	6.8	4.2	1179	62.4	63.9
11	89	1049	370	93334	7.0	4.0	626	65.4	67.0
12	20	864.6	255.0	17291	5.1	3.0	102	58.8	59.2
13	171	1142	85	195295	10.3	4.1	1763	90.2	90.3
14	211	1289	138	272029	11.6	4.2	2441	90.1	89.7
15	162	1128	83	182771	10.6	3.9	1716	93.8	93.9
16	227	1263	129	286596	11.5	4.6	2609	90.7	91.0
17	152	975.2	82.3	148224	8.7	2.9	1315	89.0	88.7
18	291	1396	147	406352	10.1	4.3	2944	73.2	72.4
19	201	929.2	149.2	186767	8.0	3.6	1601	85.0	85.7
20	288	1096	288	315780	9.4	4.6	2706	85.8	85.7
21	190	1199	284	227813	10.0	4.8	1892	83.1	83.0
22	178	1052	179	187186	8.5	3.4	1504	81.8	80.4
23	199	1026	144	204161	9.0	3.8	1790	86.9	87.7
24	159	908.5	84.4	144456	8.7	3.6	1391	96.0	96.3
25	425	1420	233	603485	12.1	4.8	5142	84.5	85.2
26	564	1335	315	752812	10.7	4.8	6061	79.2	80.5
27	75	1339	313	100399	8.0	4.2	597	60.3	59.4
28	314	1560	319	489730	8.6	3.9	2710	56.2	55.3
29	292	1193	235	348282	6.0	2.9	1754	51.2	50.3
30	167	826.3	135.4	137989	4.4	2.6	736	54.3	53.4
31	66	1111	147	73304	10.3	4.4	681	92.7	92.9
32	168	1601	88	268999	12.9	4.5	2165	80.6	80.5
33	195	1766	209	344461	15.6	4.7	3048	88.3	88.5
34	43	946.0	190.3	40678	7.6	3.9	328	80.7	80.6
35	154	864.1	96.5	133069	6.8	3.3	1045	77.6	78.5
36	58	1043	142	60514	9.3	5.8	538	89.0	88.9
37	75	1175	115	88103	7.5	3.2	565	64.4	64.1
38	88	1444	159	127071	9.7	4.8	851	66.6	67.0
39	97	1421	170	137802	8.1	3.2	790	57.2	57.3
40	90	1146	103	103116	6.6	3.2	595	57.6	57.7
41	96	1196	47	114837	6.3	3.4	605	52.4	52.7
42	286	1074	94	307113	5.5	2.9	1586	51.4	51.6
43	115	1585	251	182316	13.8	5.2	1592	87.6	87.3
44	185	1509	228	279134	13.2	4.5	2436	87.3	87.3
45	77	1422	210	109512	12.4	4.1	958	87.6	87.5
46	93	1762	154	163849	14.9	4.9	1384	85.2	84.5
47	177	1603	214	283743	13.4	5.1	2371	84.2	83.6
48	54	1415	387	76403	16.4	7.0	887	115.3	116.1
49	65	1182	168	76849	13.2	5.1	859	112.3	111.8
50	47	1050	132	49370	11.5	5.1	541	108.2	109.6
51	101	1288	211	130128	14.2	5.7	1437	110.1	110.4
52	90	1368	246	123084	14.7	5.0	1320	110.5	107.3
53	72	1194	208	86002	13.7	5.7	990	113.5	115.1

Not shown is the similar set of data for the ^{12}C, ^{13}C and $^{13}C/^{12}C$ ratios. For $^{12}C^{15}N/^{12}C^{14}N$ ratios, the numerator has been multiplied by 10,000. Note that we calculate the $^{12}C^{15}N/^{12}C^{14}N$ ratio in two ways, corresponding to the columns "Mean" and "Sum". "Means" are calculated from the derived $^{12}C^{15}N/^{12}C^{14}N$ ratio image; they represent the mean pixel ratio within an ROI in the ratio image. "Sums" are the result of the division of the sum of the ^{27}CN counts by the sum of the ^{26}CN counts within the same ROI. For a given ROI, it can be shown that any gross discrepancy between "Mean" and "Sum" indicates that the isotope incorporation is distributed at least among 2 populations within the ROI.

The data are then further reduced using the spreadsheet, statistical, graphing and modeling program Prophet (Bolt, Berenek and Newman and National Institutes of Health). The ROIs are grouped by categories - defined by anatomical recognition and/or standing-out isotope ratio values - and final results for one analytical field are generated. For example, the grouped data from the outer hair cells, (identified by ROIs 13-17) are shown in Additional Table 2. Similar tables are generated for all the group of ROIs in this field. Finally, we generate a summary table containing the consolidated statistics of the $^{12}C^{15}N/^{12}C^{14}N$ ratio values and the percentage nitrogen renewal for all the groups of ROIs. (Additional Table 3).

Additional Table 2

Regrouped data for the outer hair cells

ROI	n Pix	^{26}CN Mean	^{26}CN sd	^{26}CN Sum	^{27}CN Mean	^{27}CN sd	^{27}CN Sum	^{27}CN/^{26}CN "Mean"	Renewal "Mean"
13	171	...	...	....	...	...	...	90.2	52.0
14	211	...	...	....	...	...	...	90.1	51.9
15	162	...	...	....	...	...	...	93.8	55.5
16	227	...	...	....	...	...	...	90.7	52.5
17	152	...	...	....	...	...	...	89.0	50.8
**N**	...	5	...	5	5	...	5	5	5
**Mean**	...	1160	...	216983	10.5	...	1969	90.8	52.5
**Median**	...	1142	...	195295	10.6	...	1763	90.2	52.0
**sd**	...	125	...	59676	1.2	...	540	1.8	1.8
**C.V.**	...	0.1	...	0.3	0.1	...	0.3	0.0	0.03
**Max**	...	1289	...	286596	11.6	...	2609	93.8	55.5
**Min**	...	975.2	...	148224	8.7	...	1315	89.0	50.8

The data shown correspond to ROIs 13-17 in Additional Figure 1. Values for the mean and sum of the ^{26}CN and ^{27}CN counts are removed for clarity. The percent nitrogen renewal is calculated using the formula 100 * (Tissue $^{12}C^{15}N/^{12}C^{14}N$ ratio – control diet ratio)) / (Experimental diet ratio – Control diet ratio). N, number of ROIs; sd, standard deviation; C.V., coefficient of variation; Max, maximum value; Min, minimum value. Note that these 5 ROIs correspond to a total number of pixels of 923.

Additional Table 3
Summary of the $^{12}C^{15}N/^{12}C^{14}N$ ratios and of the percent nitrogen renewal for the different cellular structures of the cochlea

	Epon		RL		OHC		OHC N	
	$^{27}CN/^{26}CN$	% Renewal	$^{27}CN/^{26}CN$	% Renewal	$^{27}CN/^{26}CN$	% Renewal	$^{27}CN/^{26}CN$	% Renewal
**N**	6	6	6	6	5	5	1	1
**Mean**	36.5	-0.1	68.3	30.8	90.8	52.5	73.2	35.5
**Median**	36.1	-0.5	67.0	29.5	90.2	52.0	...	...
**sd**	3.0	2.9	9.2	8.9	1.8	1.8	...	-
**C.V.**	0.08	...	0.13	0.3	0.02	0.03	...	-
**Max**	41.1	4.4	85.2	47.2	93.8	55.5	...	...
**Min**	33.1	-3.4	58.8	21.6	89.0	50.8	...	...

	DC		OPC		ABM		BM	
	$^{27}CN/^{26}CN$	% Renewal	$^{27}CN/^{26}CN$	% Renewal	$^{27}CN/^{26}CN$	% Renewal	$^{27}CN/^{26}CN$	% Renewal
**N**	8	8	4	4	6	6	6	6
**Mean**	85.3	47.2	55.5	18.4	84.8	46.0	58.3	21.1
**Median**	84.8	46.7	55.3	18.1	84.5	46.1	57.4	20.2
**sd**	5.0	4.8	3.8	3.7	6.0	5.7	6.2	6.0
**C.V.**	0.06	0.10	0.07	0.2	0.07	0.1	0.1	0.3
**Max**	96.0	57.6	60.3	23.0	92.7	53.3	66.6	29.1
**Min**	79.2	41.4	51.2	14.2	77.6	39.8	51.4	14.4

	TBC t1		TBC t2	
	$^{27}CN/^{26}CN$	% Renewal	$^{27}CN/^{26}CN$	% Renewal
**N**	5	5	6	6
**Mean**	86.4	48.3	111.7	72.8
**Median**	87.3	49.2	111.4	72.6
**sd**	1.6	1.5	2.6	2.5
**C.V.**	0.02	0.03	0.02	0.03
**Max**	87.6	49.5	115.3	76.3
**Min**	84.2	46.2	108.2	69.5

RL, reticular lamina; OHC , outer hair cells; OHC N, outer hair cell nucleus; DC, Deiter cells; OPC, outer pillar cells; BM, basilar membrane; ABM, above basilar membrane; TBC t1, tympanic border cells type 1; TBC t2, tympanic border cells type 2. Note that 'Epon' corresponds to the embedding media and does not contain an excess of ^{15}N; its $^{12}C^{15}N/^{12}C^{14}N$ isotope ratio value is equivalent to the natural abundance.

In a given experiment, tens of fields are analyzed. It results in tens to hundreds of thousands of rows and several tens of columns of data values to be reduced. Data reduction of this massive amount of information would not be practical, if even possible, without the extensive although elementary

computing software that we have developed. Despite all the computational automation, however, data reduction remains at least ten times longer than the duration of analysis for an experiment.

All of the quantitative data is found within the pixels contained in the image. We want to emphasize that whatever changes we make to the appearance of the quantitative images, the counts per pixel remain strictly unchanged.

In additional figure 2a and 2b we show the $^{12}C^{14}N^-$ and $^{12}C^{15}N^-$ images from an unlabeled control mouse cochlear sample. The derived $^{13}C/^{12}C$ and $^{12}C^{15}N/^{12}C^{14}N$ isotope ratio images are shown in Figures 2c and 2d. The values of the ratios are uniform over the field regardless of their position in the image, so that the isotope ratio images 2c and 2d have no contrast.

We have drawn 27 ROI's within the image field. A portion of the statistics generated from each ROI is given in additional table 4.

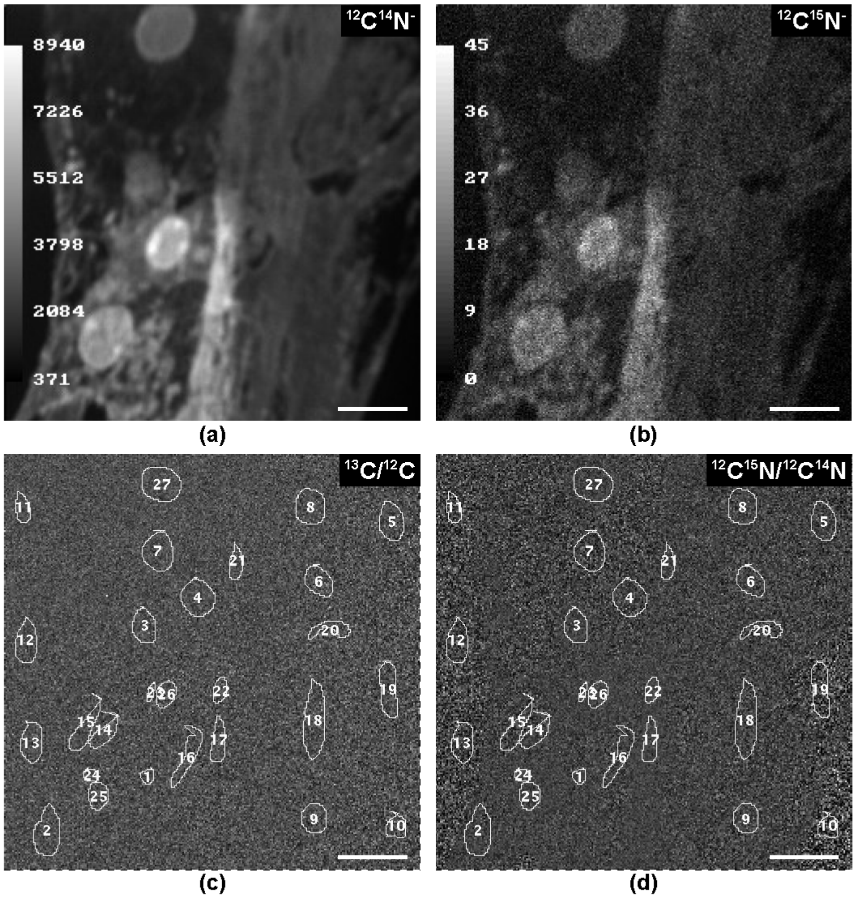

Additional Figure 2
Parallel quantitative mass image of an unlabeled control mouse cochlear section.
(a) $^{12}C^{14}N^-$ image. **(b)** $^{12}C^{15}N^-$ image. **(c)** Derived $^{13}C/^{12}C$ ratio image and **(d)** The derived $^{12}C^{15}N/^{12}C^{14}N$ ratio image with 27 ROI's manually drawn. Bar = 5 µm. 256 x 256 pixels. Acquisition: 45 minutes.

Additional Table 4
A portion of the statistical data derived from the 27 ROI's shown in Additional Figure 2.

ROI	n Pixels	^{12}C Mean	^{13}C Mean	^{26}CN Mean	^{27}CN Mean	$^{13}C/^{12}C$ "Mean"	$^{27}CN/^{26}CN$ "Mean"
1	75	1002	10.8	4371	16.1	107.9	37.0
2	422	1112	12.5	2976	11.1	112.8	37.0
3	272	1295	14.7	3271	12.2	113.5	37.1
4	400	1225	13.8	1765	6.5	112.5	36.9
5	315	769.9	8.6	1827	6.8	112.2	37.2
6	270	999.8	11.5	2109	8.0	115.5	38.1
7	390	1485	17.0	1236	4.5	114.3	36.4
8	354	1059	11.9	2609	9.9	112.9	37.9
9	253	984.8	10.7	2179	8.2	108.9	37.7
10	164	653.0	7.3	523.5	1.9	112.1	37.1
11	152	1745	19.5	924.6	3.5	111.9	37.6
12	305	1310	14.5	1018	3.7	110.8	36.4
13	295	1218	13.9	868.4	3.2	114.5	36.9
14	209	1056	12.1	4246	15.4	115.0	36.5
15	274	1169	12.9	1600	5.9	110.1	36.9
16	302	980.1	11.1	2131	7.8	113.4	36.6
17	219	905.0	10.2	6646	25.4	113.3	38.3
18	543	954.9	10.8	1972	7.5	112.9	37.9
19	344	765.2	8.4	613.1	2.3	109.4	37.2
20	174	943.0	10.8	1221	4.5	114.7	37.0
21	153	1312	15.3	2886	10.6	116.9	36.8
22	122	977.2	10.8	6485	24.6	110.2	37.8
23	52	947.1	10.8	7981	28.4	114.4	35.6
24	71	1007	11.3	6253	24.0	112.0	38.3
25	194	1019	11.4	5191	19.0	111.9	36.6
26	177	937.8	10.5	6281	23.3	111.5	37.2
27	451	1615	18.3	3380	12.6	113.4	37.2
**N**	...	...	...	...	...	27	27
**Mean**	...	...	...	...	...	112.6	37.2
**Median**	...	...	...	...	...	112.8	37.1
**sd**	...	...	...	...	...	2.1	0.6
**C.V.**	...	...	...	...	...	0.02	0.02
**Max**	...	...	...	...	...	116.8	38.3
**Min**	...	...	...	...	...	107.9	35.6

Although the mean counts among ROIs at a given mass may greatly vary the derived isotope ratio

values barely change among ROIs (CV < 2% for both $^{13}C/^{12}C$ and $^{12}C^{15}N/^{12}C^{14}N$, additional table 4). The mean ^{13}C / ^{12}C ratio value for all the ROI's are not statistically different among themselves and with the natural abundance ratio (1.12 %) . The mean $^{12}C^{15}N$ / $^{12}C^{14}N$ ratio for all the ROI are not statistically different among themselves, and their mean 0.372% is very close to the natural abundance ratio, 0.367%; yet, because the data is so tight, the values are significantly different ($p < 0.05$). This is very likely the result of a residual slight imbalance after tuning of the detectors measuring the $^{12}C^{15}N$ and $^{12}C^{14}N$ secondary ion current.

Note that in this control sample, both the mean $^{13}C/^{12}C$ and $^{12}C^{15}N/^{12}C^{14}N$ isotope ratio values are independent of the location of the ROI within the image field. In ^{15}N-labeling experiments the value of the $^{12}C^{15}N/^{12}C^{14}N$ isotope ratios is depending upon the biological object and thus vary with the position in the analyzed field. This underscore the importance of the $^{13}C/^{12}C$ ratio image in providing an internal control that the image field is balanced.
