# Supplementary material for: High-resolution quantitative imaging of mammalian and bacterial cells using stable isotope mass spectrometry
Source: J Biol. 2006 Oct 5;5(6):20. doi: 10.1186/jbiol42 (PMC1781526; doi:10.1186/jbiol42)
Supplement: Additional data file 4 — The relationship between the brightness of an image and the number of counts [file jbiol42-s4.pdf]

Additional File 4: Relationship between the brightness of an image and the number of counts

Let us consider a same field analyzed with two different acquisition times in the ratio of 1 to 10, for example, and displayed using a linear gray scale of 256 levels (as it is usually done, although the human eye cannot distinguish more than approximately 90 shades of gray) between 0 counts and Nmax (from deep black to saturated bright white). Nmax refers to the maximum of counts in a pixel within the field of view. Depending upon the value of Nmax, the two pictures may look quite different in the display. The low-Nmax picture may exhibit statistical fluctuations, whereas the high Nmax may appear 'saturated' in some parts of the field, leading to the suppression of interesting details in the display. In this case it is often useful to introduce a threshold Nmin (or Nmax) and to display the number of counts within a gray scale from black set at Nmin to white set at Nmax. Depending upon the value chosen for Nmin (or Nmax), the gray scale is more or less expanded and can be optimized for showing details of interest. An image with more counts can then appear less 'bright' than an image with low counts. Of course, one should be very cautious in interpreting the pictures; the 'true' information is given by the number of counts recorded in each pixel.
