# Supplementary material for: High-resolution quantitative imaging of mammalian and bacterial cells using stable isotope mass spectrometry
Source: J Biol. 2006 Oct 5;5(6):20. doi: 10.1186/jbiol42 (PMC1781526; doi:10.1186/jbiol42)
Supplement: Additional data file 5 — A comparison of MIMS with autoradiography [file jbiol42-s5.pdf]

Additional File 5: MIMS analysis vs. ^{14}C Autoradiography

$$N=N_0e^{-\gamma t}$$

where N_0 is the number of atoms at time 0

and N number of atoms after radioactive decay time t

γ = decay constant; $\gamma = 0.693/T_{1/2}$

^{14}C $t_{1/2}$ = 5715 years $\gamma = 2.3\text{x}10^{-10}$ per minute

Number of radioactive ^{14}C atoms in 1 µC :

1 µC =2.22 x 10^6 dpm

$N = N_0\exp(-2.3\text{x}10^{-10})$

After 1 minute: $N = N_0 - 2.22\text{x}10^6$

$N_0 - (2.22\text{x}10^6) = N_0\exp(-2.3\text{x}10^{-10})$

$N_0 = 9.56\text{x}10^{15}$

Fraction of ^{14}C atoms disintegrated in 1 minute per µC:

$2.22\text{x}10^6 / 9.56\text{x}10^{15}$

Thus, even if only 1‰ of the ^{14}C atoms were sputtered and if only 1% of the sputtered atoms were ionized,

the number of secondary $^{14}C^-$ ions would be $9.56\text{x}10^{10}$

and **the efficiency of MIMS would be**

$9.56\text{x}10^{10} / 2.22\text{x}10^6$

or **approximately $5\text{x}10^4$ fold higher than that of autoradiography.**
