# Supplementary material for: High-resolution quantitative imaging of mammalian and bacterial cells using stable isotope mass spectrometry
Source: J Biol. 2006 Oct 5;5(6):20. doi: 10.1186/jbiol42 (PMC1781526; doi:10.1186/jbiol42)

Additional File 6: Flat Top Peaks in Mass Spectra

In first approximation, the shape of the recording showing a mass spectrum depends upon the width of the mass lines, the width of the mass selection slit and the separation of the mass lines in the focal plane. In isotopic work it is usual to have a mass selection slit larger than the mass line being selected in order to prevent the signal variations that can arise from the possible drifts in the mass line position. In consequence, the mass line appears as a peak with a flat top in the recording. Two adjacent lines may be completely separated and appear with no valley between their flat topped peaks in the recording.

From the figure below, it can be seen that if Lw = Ls and Ls = 2xLm, a given mass line can be isolated from the adjacent one although the conditions are such that there will be no valley between the peaks in the mass spectrum recording.

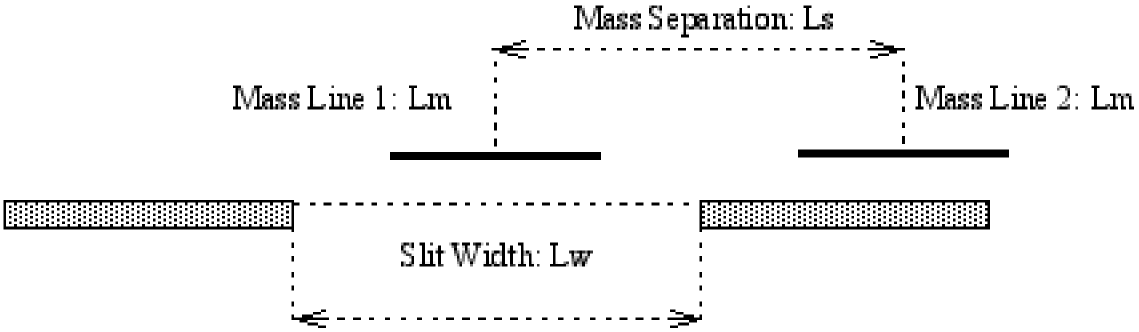

Supplement: Additional data file 6 — An explanation of the shape of mass line peaks in MIMS [file jbiol42-s6.pdf]
