# Supplementary material for: High-resolution quantitative imaging of mammalian and bacterial cells using stable isotope mass spectrometry
Source: J Biol. 2006 Oct 5;5(6):20. doi: 10.1186/jbiol42 (PMC1781526; doi:10.1186/jbiol42)
Supplement: Additional data file 7 — More details on Figure 2e [file jbiol42-s7.pdf]

Additional File 7: Supplementary data to Figure 2e

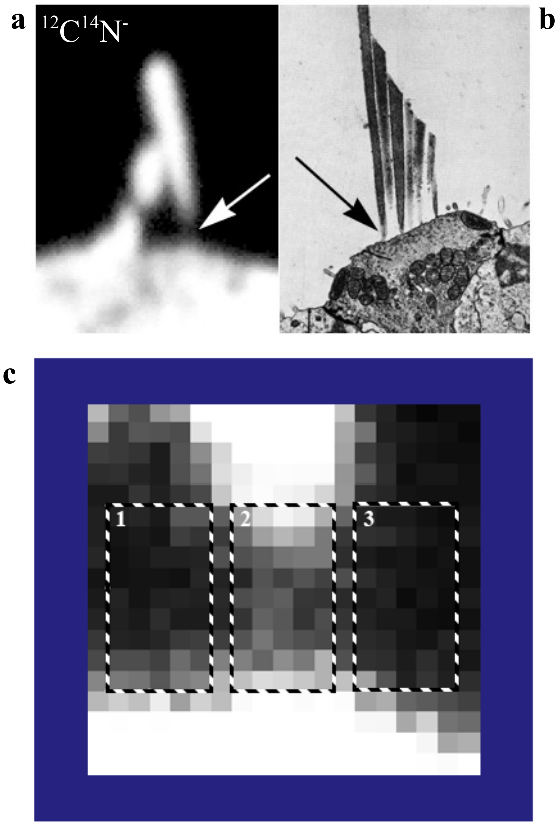

d COMPARISON OF $^{12}C^{14}N^{-}$ COUNTS IN ROI 1,2 AND 3

<table>
<tr><th>ROI</th><th>n</th><th>Mean</th><th>SD</th><th>t</th><th>t</th></tr>
<tr><td>1</td><td>45</td><td>122.5</td><td>47.1</td><td rowspan="2">8.1</td><td></td></tr>
<tr><td>2</td><td>45</td><td>212.1</td><td>57.1</td><td rowspan="2">11.5</td></tr>
<tr><td>3</td><td>45</td><td>93.6</td><td>39.1</td><td rowspan="2">3.2</td></tr>
<tr><td>1</td><td>45</td><td>122.5</td><td>47.1</td><td></td></tr>
</table>

(a) Region of figure 2e including stereocilia, S. The arrow points to the base of stereocilia, 'BS' in figure 2e, where it inserts in the cuticular plate.
(b) Electron micrograph showing the stereocilia of a hair cell. The arrow points to the base of the stereocilia which is narrower and less electron-dense than the bulk of the stereocilia [45].
(c) A zoomed in image of **(a)** at the level of the base of stereocilia.
(d) Comparison of the $^{12}C^{14}N^{-}$ counts in the three regions of interest (ROI1, ROI2, and ROI3) that are boxed in (c); they are all 5 x 9 pixels. The three ROI's are separated by 23.4 nm. The area included in each ROI is 0.025 μm^2. n is the number of pixels. 'Mean'

indicates the number of $^{12}C^{14}N$ counts per pixel, after the counts from the epon background have been subtracted.

ROI2 contains a mean number of $^{12}C^{14}N$ counts that is significantly higher than those of ROI1 and ROI3 but much lower than that over the stereocilia core (mean = 1,873 counts; not shown). This indicates that MIMS can directly detect the presence of nitrogen-containing material, (likely protein), barely visible on the image. At this location the stereocilia taper above their insertion into the cellular apex and, in the bullfrog, the microfilaments of the stereocilia core, and thus the approximate amount of protein, decrease in number by a factor of approximately 20 (see Jacobs, R.A. And A.J. Hudspeth, *Ultrastructural correlates of mechanoelectrical transduction in hair cells of the bullfrog's internal ear.* Cold Spring Harb Symp Quant Biol, 1990. **55:** p.547-61)
